# Developing hydrogels for gene therapy and tissue engineering

**DOI:** 10.1186/s12951-024-02462-z

**Published:** 2024-04-15

**Authors:** Chunyu Su, Dini Lin, Xinyu Huang, Jiayin Feng, Anqi Jin, Fangyan Wang, Qizhuang Lv, Lanjie Lei, Wenjie Pan

**Affiliations:** 1 Key Laboratory of Artificial Organs and Computational Medicine in Zhejiang Province, Institute of Translational Medicine, Zhejiang Shuren University, Hangzhou, 310015 China; 2 The Third Affiliated Hospital of Wenzhou Medical University, Wenzhou, 325200 China; 3 College of Biology & Pharmacy, Yulin Normal University, Yulin, 537000 China

**Keywords:** RNA, Exosome, Hydrogel, Gene therapy, Tissue engineering

## Abstract

Hydrogels are a class of highly absorbent and easily modified polymer materials suitable for use as slow-release carriers for drugs. Gene therapy is highly specific and can overcome the limitations of traditional tissue engineering techniques and has significant advantages in tissue repair. However, therapeutic genes are often affected by cellular barriers and enzyme sensitivity, and carrier loading of therapeutic genes is essential. Therapeutic gene hydrogels can well overcome these difficulties. Moreover, gene-therapeutic hydrogels have made considerable progress. This review summarizes the recent research on carrier gene hydrogels for the treatment of tissue damage through a summary of the most current research frontiers. We initially introduce the classification of hydrogels and their cross-linking methods, followed by a detailed overview of the types and modifications of therapeutic genes, a detailed discussion on the loading of therapeutic genes in hydrogels and their characterization features, a summary of the design of hydrogels for therapeutic gene release, and an overview of their applications in tissue engineering. Finally, we provide comments and look forward to the shortcomings and future directions of hydrogels for gene therapy. We hope that this article will provide researchers in related fields with more comprehensive and systematic strategies for tissue engineering repair and further promote the development of the field of hydrogels for gene therapy.

**Graphical abstract:**

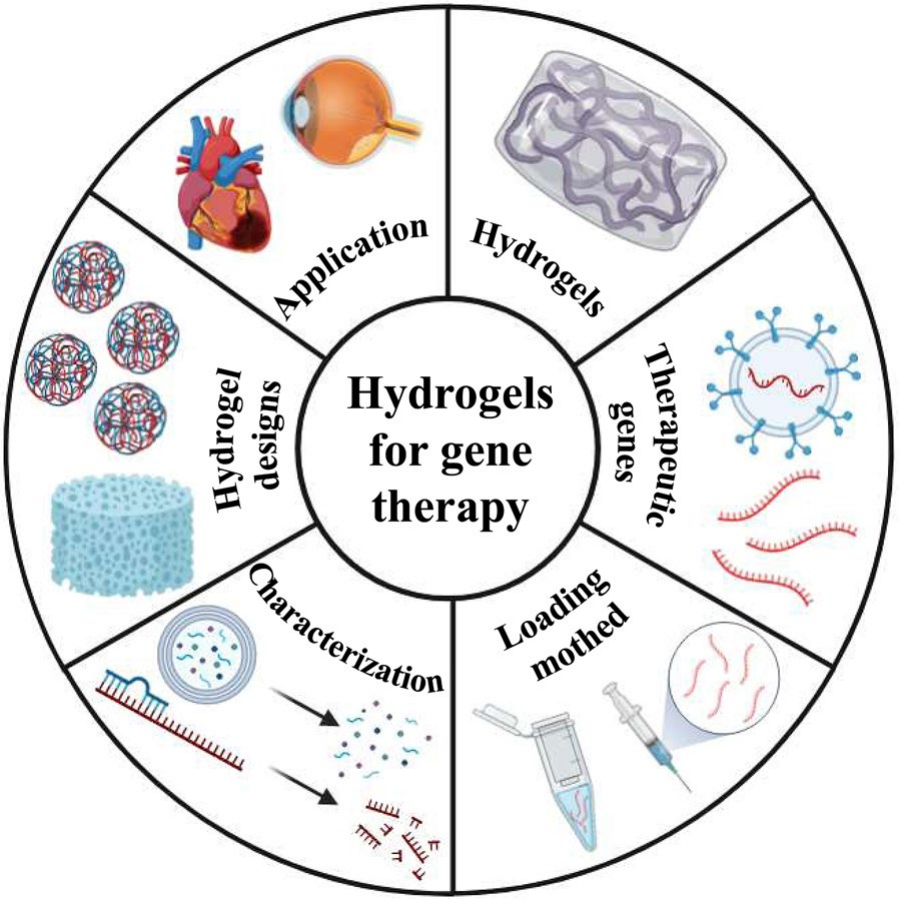

## Introduction

The human body inevitably encounters tissue damage owing to occurrences such as car accidents, burns, and accidental damage. Part of the persistent infection caused by chronic injury will lead to uncontrolled immune regulation. Infected bone defects and traditional surgical resection of tumors are prone to recurrence, while tissue transplantation will cause immune rejection, among other issues, which will further aggravate the patient’s symptoms and even lead to death [1]. Moreover, the human body’s self-repair ability is very limited; therefore, regulating and invoking gene therapy is a very promising method to further improve the therapeutic effect. Gene therapy is the introduction of genes into cells to up-regulate the expression of target genes to treat or repair tissue defects [2, 3]. It exhibits high specificity, low immune rejection, and durability; it is widely used in the treatment of cystic fibrosis, hereditary retinopathy, multiple sclerosis, bone defect repair, and cancer [4, 5].

Direct injection of gene drugs has shortcomings, such as rapid degradation, easy off-target effects, and low bioavailability. The hydrogel is an excellent class of carrier material; it is a porous hydrophilic three-dimensional network structure with good biocompatibility, biodegradability, and water retention. It can improve the stability of therapeutic genes, which can enhance cell adhesion, transfection efficiency, and targeting precision. Hydrogels have good mechanical properties and are less invasive, injectable, and easy to modify and customize, which is conducive to the efficient delivery of gene therapy [6]; thus, the combination of gene therapy with hydrogels presents a promising avenue for tissue damage repair.

An increasing number of gene therapy hydrogels for tissue engineering have been developed. However, several unresolved problems remain [7, 8]. For example, the poor mechanical properties and stability of some gene therapy hydrogels can lead to immune reactions and toxic effects; their safety and effectiveness are not guaranteed. Poor transfection efficiency may hinder the efficient delivery of gene drugs. To fully realize the potential of gene therapy hydrogels in tissue engineering repair, continual optimization of the key steps is required such as more efficient loading of genes into the hydrogel, improving the stability and activity of genes in vivo, and refining the efficiency of gene transfection.

Despite these challenges, considerable progress has been made in the development of gene therapy hydrogels. Therefore, this review initially introduced the classification of hydrogels and their cross-linking methods. Subsequently, we provide a detailed overview of the types and modifications of therapeutic genes, followed by a detailed discussion on the loading of therapeutic genes in hydrogels and their characterization features, a summary of the design of hydrogels for therapeutic gene release, and an overview of their applications in tissue engineering. Finally, we provide comments on the shortcomings and future directions of hydrogels for gene therapy. We envisage that this article will provide researchers in related fields with more comprehensive and systematic strategies for tissue engineering repair to promote the development of the field of hydrogels for gene therapy.

## Hydrogels

**Fig. 1:**
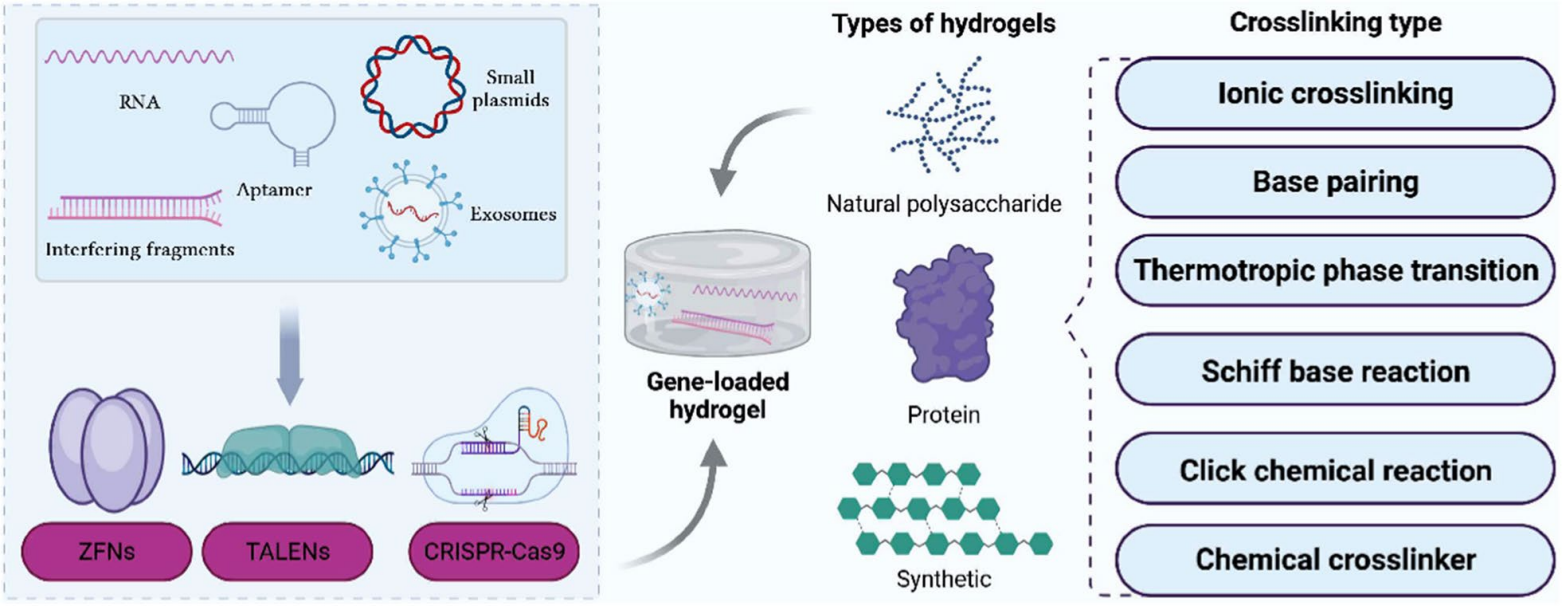
Types of hydrogels and their crosslinking modes and therapeutic gene types and their modification strategies. Created with BioRender.com. (Agreement number: EC26NU6OOQ)

Hydrogels can absorb large amounts of water, have high water swelling, and maintain their original structure without dissolution, even after substantial expansion. This attribute is advantageous to improve cell adhesion and transfection efficiency and promote gene stability by preventing degradation [9]. Currently, hydrogels are less invasive, injectable, and customizable, and are suitable for efficient delivery of gene therapy drugs, showing great potential for tissue repair (Fig. 1). In the following section, we explore the types and properties of hydrogels. Furthermore, we summarize the commonly used crosslinking methods, with detailed descriptions (Table 1).

**Table 1:** Classification and properties of hydrogels and their crosslinking modes

Type	Species	Characteristic	Crosslinking method
Physical crosslinking	Sodium alginate	Biocompatibility, non-toxicity, biodegradability	Ionic crosslinker [10–12], chemical crosslinker [10]
	Chitosan	Bacteriostatic, pressure resistant, easily modified	Photo crosslinker [13], Michael addition [14], enzyme crosslinker [15]
	Hyaluronic acid	Personalized customization, long-term effectiveness, biocompatibility	Chemical crosslinker [16]
	Agarose	Antiadhesion, heat reversible, biocompatibility	Thermotropic phase transition [17, 18]
	Chondroitin sulfate	Pain relief, promote cell growth, promote cartilage regeneration	Schiff base reaction [19, 20]
	Glucan	Blood plasma substitutes, promote cartilage regeneration	Schiff base reaction [21], click chemical reaction [22]
Chemical crosslinking	Gelatin	Thermo-reversible, inexpensive, easy to process	Thermotropic phase transition [23–25]
	Collagen	Promote cartilage regeneration	Chemical crosslinker [26]
	Silk proteins	Mechanical property, biocompatibility, degradability	Photo crosslinker [27]
	Sericin protein	Anti-wrinkle, high elasticity, tensile	Enzyme crosslinker [28], photo crosslinker [29]
	Albumin	Detoxification, antioxidant, biocompatibility, solubility	Chemical crosslinker [30, 31]
Synthetic hydrogels	NIPAM	Heat sensitivity, degradability, smart-responsive materials	Click chemical reaction [22], thermotropic phase transition [32]
	PEG	Solubility, softener, antistatic agent	Michael addition [33]
	Acrylic acid	Hydrophilicity	Photo-cross-link [34], free-radical polymerization [35]
	Acrylamide	Cytocompatibility, responsiveness, cell adhesion	Chemical crosslinker [36, 37]

*NIPAM* N-isopropyl acrylamide, *PEG* polyethylene glycol

### Classification of hydrogels

#### Natural polysaccharide hydrogels

Sodium alginate (SA) is a by-product of the extraction of iodine from brown algae, which is formed by the connection of α-L-guluronic and β-D-mannuronic [38]. Its hydrogels exhibit good biocompatibility, biodegradability, stability, and high viscosity, which can provide a moist environment for wound repair. However, their lack of mechanical strength limits their application, and intermolecular crosslinking can be induced by the addition of ionic crosslinking agents, for example, Ca^2+^ and Al^3+^, which improves the mechanical strength [39]. The applications of SA hydrogel-based gene therapy are well established. For example, Li et al. [40] used the 45S5 Bioglass® (BG)/SA hydrogel to encapsulate small interfering RNA (siRNA) from matrix metalloproteinase 9 (MMP9), which can directly inhibit the overexpression of MMP-9 in cells, thus reducing inflammation, accelerating traumatic angiogenesis, and promoting the synthesis of collagen and fibroblast extracellular matrix proteins (ECMs). Owing to the high viscosity of the SA hydrogel, it covers and adheres more firmly to and better protects the damaged tissue area, especially in the treatment of skin wounds [39].

Chitosan is derived from the partial deacetylation of chitin, an alternate link between N-acetylglucosamine and glucosamine [40]. Chitosan hydrogels are bacteriostatic, compression-resistant, have good adhesion, and can be easily chemically modified. Through modification, this material can significantly improve the binding capacity and delivery efficiency to genes, thus promoting tissue wound healing more effectively [41]. Cai et al. [15] used MMP-2 to modify carboxymethyl chitosan (CMCS-AGE) and prepared a hydrogel by loading siRNA complexes that target transforming growth factor-β1 (TGF-β1). The resulting hydrogel upregulated MMP-2 expression in tendon tissues, allowing controlled release of siRNA, which largely reduced the negative effects on tendon healing. Gene therapy often results in decreased therapeutic effects owing to off-target effects and low transfection efficiency. To address these issues, multifunctionality has emerged as a strategy. Chitosan can easily be chemically modified, significantly enhancing cell targeting, which greatly improves the targeting and efficiency of gene therapy. Therefore, the synergistic effect of pluripotent chitosan and gene therapy may be an important topic for future research [41].

Hyaluronic acid (HA) can be obtained by microbial fermentation and extraction of animal tissues. It comprises alternately attached N-acetylglucosamine and glucuronic acid [42]. Hyaluronic acid hydrogels have good biocompatibility and pH response and can be customized according to the size, shape, position, and other factors of the wound of the patient [43]. Sun et al. [16] prepared a THH-3/Exos-microRNA (miRNA) 24-3p hydrogel composite. The gel promotes migration and accelerates the rate of wound healing in rabbit corneal epithelial cells. An inhibitory effect on keratitis and corneal fibrosis is also evident in animal burn models. Its functionality is outstanding compared with those of other materials owing to the natural repair effect of HA hydrogels on tissues, although the mechanical properties of these hydrogels are poor, and the degradation rate is difficult to control; therefore, HA hydrogels require further improvement. This might be achieved by adjusting the concentration of cross-linking agents, the timing of the cross-linking reaction, or the conditions to improve their mechanical properties. Moreover, the modulation of the degradation rate could be improved by intelligent responsive design [43].

Agarose (AG) is a linear polysaccharide found in red algae, comprising alternating arrangements of 1,3-linked β-D-galactose and 1,4-linked 3,6-endo-ether-L-galactose [44]. Agarose hydrogels possess exceptional biocompatibility, antiadhesion, degradability, and thermo-reversible properties, allowing them to protect genes from the external environment and enhance the efficiency and stability of gene delivery [17]. siRNA targeting the mRNA encoded by the *Col1a1* gene (Col1a1 siRNA) is a crucial regulatory substance for invasive chondrocyte therapy. Chondrocytes transfected with Col1a1 siRNA and encapsulated in an AG hydrogel effectively promote long-term proliferation and expression of the corresponding proteins [17]. The AG hydrogel has low immunogenicity and mitigates the risk of immune rejection, rendering it indispensable for gene therapy after tissue damage. However, its limitation lies in the inability to specifically target cells for gene transfection, particularly impeding their application in tissue traumas that require high specificity in gene therapy. Thus, future advances in cell-specific improvements are crucial for the continued development of AG hydrogels [17].

Chondroitin sulfate (CS) is widely distributed in animal cartilaginous tissues and comprises N-acetylgalactose linked to D-glucose via a 1,4-glucosidic linkage [45]. Chondroitin sulfate hydrogels are biocompatible and degradable, promote cell adhesion and proliferation, and reduce serum cholesterol and triglyceride levels in patients with hyperlipidemia, thus decreasing the incidence of coronary heart disease [46]. Zhang et al. [47] prepared hydrogels with strong adhesion and biocompatibility by modifying CS and alginate-dopamine with enbucrilate. The loading of exosomes (Exos) efficiently promotes the proliferation, differentiation, expansion, and migration of bone marrow mesenchymal stem cells (BMSCs). In situ injection of this hydrogel into the rat patellofemoral groove resulted in the recruitment of BMSCs into newborn cartilage under the guidance of the chemokine signaling pathway. This accelerated extracellular matrix (ECM) remodeling and regeneration of cartilage defects. Although the CS hydrogel protects therapeutic genes from degradation factors in vivo, it lacks efficient gene transfection capabilities, causing the additional use of viral vectors or other gene transfection reagents to enhance entry into cells and expression levels.

Dextran derived from the cell walls of cereals such as wheat, oats, and yeast constitutes homopolysaccharides with glucose units linked by glycosidic bonds and serves as a substitute for plasma in medicine [48]. Dextran hydrogels exhibit good biocompatibility, biodegradability, high gene transfection efficiency, and immunoisolation effects, reducing the immune response of the body and protecting therapeutic genes during gene transfection. The hydrogel obtained by coupling MAES, dextran, and siRNA exhibits good degradability and tunability, continuously presents siRNA to enhance osteogenic differentiation of encapsulated human mesenchymal stem cells (hMSCs), and exhibits controlled cell adhesion and outstanding siRNA delivery capacity [49]. Although dextran hydrogels are not considerably toxic to cells, residual substances (including incompletely polymerized monomers) are present during preparation, which can have a toxic effect on cells. Thus, surface modification and the addition of bioactive substances are necessary to further the gene therapy applications of chitosan hydrogels [49].

#### Protein hydrogels

Gelatin is a product of collagen breakdown composed of proline, hydroxyproline, and oxyribonucleic acid linked by peptides or hydrogen bonds [50]. Photo crosslinking promotes the crosslinking of gelatin and methacryloyl anhydride under UV irradiation to form GelMA, resulting in the controlling the release or localization of therapeutic genes/growth factors, and improving gene therapy accuracy. Gelatin hydrogels exhibit good cell adhesion, biocompatibility, and processing properties, and can be prepared into different shapes and sizes of hydrogels, which makes them more suitable for different damaged tissue repairs [51]. Pan et al. [52] prepared miR-29b/gold nanoparticle (AuNP) hydrogel composites using 3D printing technology, which can mimic the bone healing process in vitro, achieve a slow release of miRNAs, and gradually replace the gel surface with new tissue, which is suitable for bone repair. Research on gelatin hydrogels has matured, and the gelatin material after photo crosslinking can better load the therapeutic drug, which is suitable for tissue trauma. However, excessive UV irradiation can lead to a mutation of the gene therapy drug, which will reduce the therapeutic effect. Future studies should investigate ways to reduce the number of mutations.

Collagen is the most abundant class of proteins in mammals and is widely distributed in the connective tissues of animals; it comprises three intertwined peptide chains [53]. Collagen modification by photo crosslinking can significantly increase its tensile strength and tensile coefficient and reduce the elongation at break, which can better meet the needs of gene therapy; however, the mutation and other problems caused by photo crosslinking modification must be emphasized. Collagen hydrogels exhibit good bioactivity, biocompatibility, and low immunogenicity, and maintain stability in vivo without causing strong immune and inflammatory reactions, with improved efficacy and safety. Weißenberger et al. [54] used an adenoviral vector containing BMP-2 cDNA and TGFB 1 to modify hMSCs and placed them in a type I collagen hydrogel. Within the cartilage medium, the gel significantly promoted glycosaminoglycan (GAG) synthesis and effectively induced cartilage formation in mesenchymal stem cells (MSCs). The low immunogenicity of collagen hydrogel is excellent for organismal safety and renders it more suitable as a gene delivery vector, although the degradation rate of collagen hydrogel is difficult to control. The future degradation rate of collagen hydrogels might be improved by surface modification, addition of cross-linking agents, and introduction of specific enzymatic sequences [54].

Silk proteins (SF) are macromolecular fibrous proteins of silkworm silk comprised of several protein subunits, including α-serin and β-serin subunits [55]. Silk protein hydrogels show good biocompatibility, degradability, mechanical properties, and ease of modification, integrate well with host tissues, and reduce inflammatory reactions and immune rejection during gene therapy [27]. Han et al. [56] prepared hydrogels by coupling silk gum (SS) with SF and used them to encapsulate human umbilical cord MSC-derived exosomes (UMSC-Exos). The wound closure assay and the in vitro and in vivo inflammatory responses showed that the hydrogel had a significant effect on improving skin fibroblast viability, reduced TNF-α secretion, and macrophage inflammatory response, and accelerating vascular regeneration and wound healing efficiency. The synergistic effect of multifunctionalized SF hydrogels with gene therapy presents a promising research avenue for introducing additional therapeutic functions or improving targeting, rendering them ideal gene delivery carriers, and improving the relevance and efficiency of gene therapy.

Sericin is a globulin produced by silkworms during the production process and comprises amino acids such as serine, aspartic acid, and glycine [57]. Sericin hydrogels have good elasticity, tensile strength, biocompatibility, safety, and mechanical properties, which can enhance the efficacy and safety of gene therapy and repair damaged tissues. Kanazawa et al. [29] used a sericin hydrogel to load siRNA targeting RelA (anti-RelA siRNA) to achieve sustained release of siRNA in vitro, which was not degraded for a long time in the rat knee joint. The treatment of collagen-induced arthritis (CIA) mice with a sericin hydrogel containing anti-RelA siRNA significantly improved the incidence rate, clinical severity, and thickness of the knee joint compared with the control group. Furthermore, sericin has natural anti-infective properties and can be used for wound suturing. Moreover, sericin hydrogels have good mechanical strength, which can provide sufficient support to satisfy the strength requirements for tissue engineering, improve the efficacy and stability of gene therapy, and accelerate the healing and functional recovery of tissues.

Albumin is a predominant protein in human plasma; it is synthesized as prealbumin and plays a role in maintaining plasma osmolality, transport, detoxification, and anti-inflammatory effects [58]. Albumin hydrogels usually exhibit good biocompatibility, degradability, adhesion, and mechanical properties, which significantly reduce adverse reactions when fused with host tissues. However, its lack of bioactivity limits its application, which can be enhanced by the addition of bioactive molecules, surface functionalization, gene transduction, and complexation with other biomaterials [30]. Chen et al. [31] loaded adipose stem cell-derived Exos onto Ag@bovine serum albumin (BSA) nanoflowers (Exos-Ag@BSA NFs) to form a protective delivery structure that precisely released Exos to remodel the trauma environment, protected Exos from oxidative denaturation and elimination of bacteria, and induced apoptosis of damaged oxidized cells. It can accelerate the rate of diabetic wound healing by promoting angiogenesis and epithelial regeneration, collagen deposition, tissue granule formation, and blood perfusion. The albumin hydrogel adheres more firmly to the damaged area, delays gene therapy, and greatly improves tissue wound repair to exhibit a good mucoadhesive effect.

#### Synthetic hydrogels

N-isopropyl acrylamide (NIPAM) is an acrylamide derivative monomer that is used to prepare poly(N-isopropyl acrylamide) (PNIPAM) [59]. NIPAM hydrogels have better biocompatibility, degradability, and thermal sensitivity and are widely used to prepare smart response materials capable of localized drug release at specific sites in gene therapy [60]. Yang et al. [61] used layered double hydroxides (LDHs) to modify PNIPAM to prepare hydrogels that are non-inhibitory to cells, can be used as thermotropic telescopic injection carriers for siRNA transduction, and can specifically modulate several factors in cartilage tissue, thus realizing in vivo gene therapy. Moreover, the PNIPAM composite hydrogel effectively inhibited degenerative factors and supported the cells. Although NIPAM hydrogels exhibit excellent thermal sensitivity, they do not have a good swelling rate. This requires alteration of the crosslinking agents and initiators or the addition of bioactives to improve their deficiencies and meet the needs of gene therapy.

**Fig. 2:**
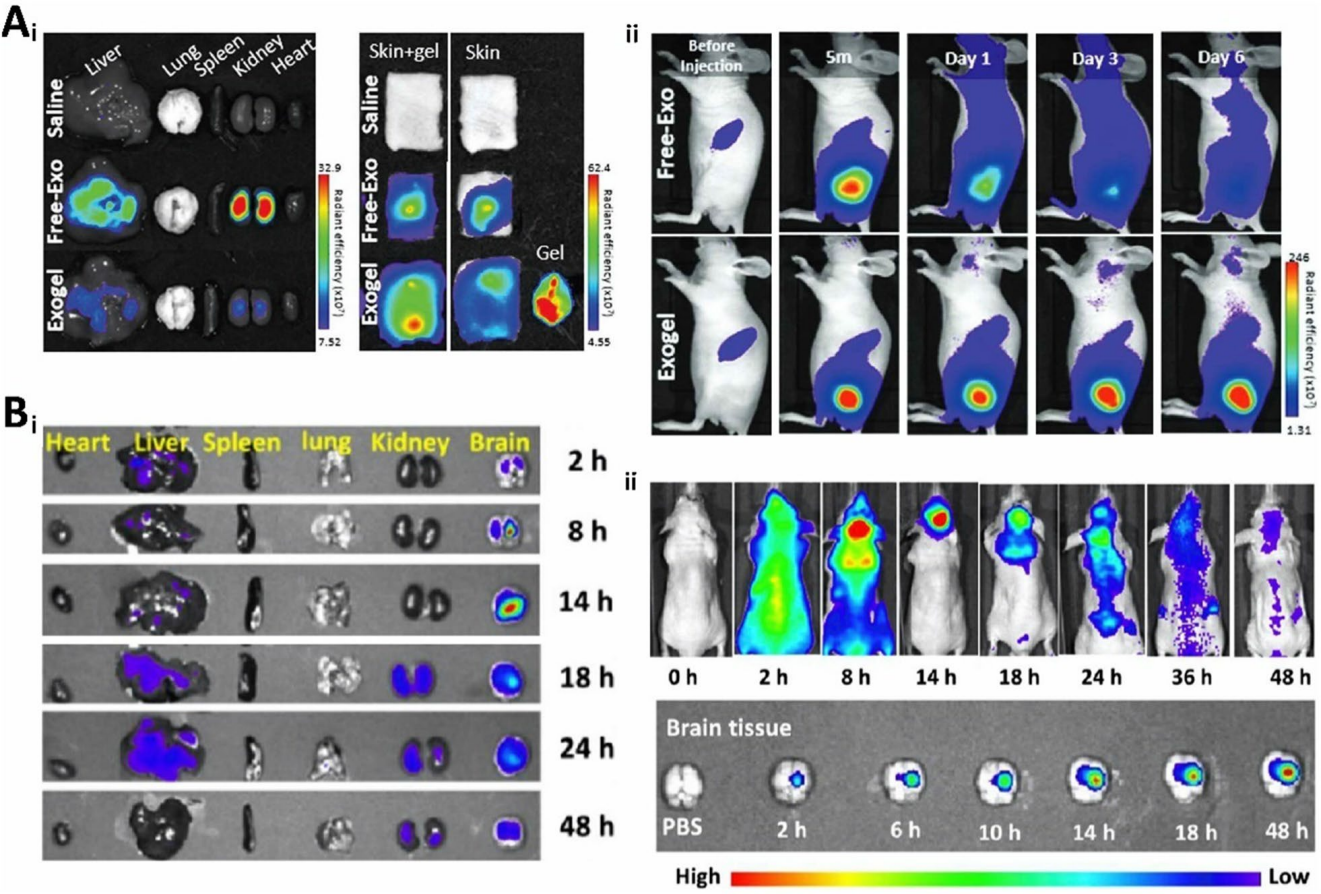
In vivo localization and distribution of therapeutic genes. **A** In vivo localization of M2-Exogels. (i) Ex vivo fluorescent images of some rat organs; (ii) Fluorescent images of biodistribution of cy5.5-labeled M2-Exos in BALB/c nude mice. (Reproduced with permission from Ref. [64], Copyright 2022, John Wiley and Sons). **B** Distribution diagram of FAM-siRNAs. (i) Distribution of FAM-siRNA in selected organs; (ii) Fluorescent images of FAM-siRNA after in situ transplantation in nude mice. (Reproduced with permission from Ref. [66], Copyright 2023, American Chemical Society)

Polyethylene glycol (PEG) is a commonly used reagent by gradual addition polymerization of ethylene glycol with ethylene oxide [62, 63]. Polyethylene glycol hydrogels have good biocompatibility, biodegradability, and processing properties, as well as good mechanical strength and elasticity, which protect therapeutic genes from the complex environment of the body and improve the rate of tissue engineering repair (Fig. 2A) [64]. Wang et al. [65] coupled difunctionalized polyethylene glycol, coralline hydroxyapatite, glycol chitosan, and SF into a hydrogel and loaded human umbilical cord mesenchymal stem cells (hucMSC)-derived Exos. The hydrogel has outstanding biocompatibility, stability, and mechanical properties that can effectively accelerate vascular regeneration and promote BMP-2 deposition, osteogenic differentiation of mouse osteoblast progenitor cells (mOPCSs), and proliferation and migration (Fig. 5B). As PEG hydrogels have good processing properties, they can be prepared according to the target shape and size, which meets the customization needs of different gene therapy applications and broadens the selection and application of gene therapy materials.

Acrylic acid (AA) is the simplest unsaturated carboxylic acid, which can be prepared by the oxidation of acrolein or the hydrolysis of acrylonitrile [67]. It is known for its corrosive, unstable nature, and susceptibility to easy polymerization. Acrylic acid hydrogels offer controllable biodegradability, ease of processing, and customization, rendering them suitable for creating materials with diverse properties to address various gene therapy applications. Nuhb [68] copolymerized tri(ethylene glycol)methyl ether methacrylate with pentafluorophenyl methacrylate via RAFT and subsequently converted it into nanohydrogel particles using an amine-containing crosslinking agent for siRNA delivery. Champeau et al. [69] developed hydrogels using polyacrylic acid-coupled PEO-PPO-PEO (F127). These hydrogels promote the expression of IL-10, SDF-1, IGF-1, and TGF-β, leading to increased fibrous collagen tissue and angiogenesis, ultimately aiding in wound repair. However, AA hydrogels exhibit instability and are susceptible to factors such as temperature, pH, and ionic strength, affecting the delivery of therapeutic genes and resulting in abnormal cell growth and proliferation in gene therapy. Strategies for enhancement include UV irradiation, the use of chemical crosslinking agents (such as ethylene glycol diacrylate), and the addition of nanoparticles to enhance mechanical strength and stability. Furthermore, the presence of acetone in AA hydrogels (which possess some toxicity) can be mitigated by introducing biocompatible groups or molecular chains, optimizing the polymerization reaction conditions, and increasing the amount of crosslinking agent.

Acrylamide is an unsaturated linear polymer that serves as a crucial water treatment agent, thickener, and flocculant synthesized by catalytic hydration. Acrylamide hydrogels exhibit commendable biocompatibility, gene transfection ability, and facile chemical modification, effectively shielding genes from in vivo degradation factors and significantly improving transfection efficiency. Chang et al. [70] developed a DNA-based acrylamide hydrogel microcapsule to achieve miRNA-responsive effects for the detection of cancer-associated miRNAs. To meet clinical requirements, they combined strand displacement polymerization/nicking amplification machinery (SDP/NA) with a hydrogel sensor, enhancing the sensitivity of miRNA detection. However, polyacrylamide hydrogels are typically cytotoxic, may induce immune reactions upon repeated use, and possess a slow degradation rate, potentially affecting the healing speed of gene therapy tissue engineering. Therefore, more research is imperative to address these limitations.

### Crosslinking of hydrogels

Crosslinking is essential to enhance hydrogel functionalization, alter surface properties, and improve characteristics. Hydrogel crosslinking falls primarily into two categories: physical and chemical. Physically cross-linked hydrogels are pure and exhibit minimal side effects, high elasticity, and plasticity. In contrast, chemically cross-linked hydrogels offer mechanical stability, adjustability, and biocompatibility. The subsequent sections detail different types of crosslinking, presenting a comparison of their advantages and disadvantages (Table 2).

**Table 2:** Comparing the advantages and disadvantages of hydrogel crosslinking methods

Type	Crosslinking method	Principle	Advantage	Disadvantages
Physical crosslinking	Ionic crosslinking	Ionic bond	Improved thermal stability and mechanical strength	Limited biocompatibility, and sensitive to environmental factors
	Base pairing	Hydrogen bond, double-stranded interaction	Strong recognition ability, accuracy, sensitivity	Poor mechanical properties, and complex preparation process, and high cost
	Thermotropic phase transition	Temperature change	Low toxicity, in situ curing	Low mechanical properties, temperature sensitive
	Molecular-specific recognition	Van der Waals forces and electrostatic interactions	High specificity and sensitivity, good stability, and good biocompatibility	Precise control of the preparation process, and sensitive to environmental factors
Chemical crosslinking	Schiff base reaction	Nucleophilic addition elimination, lone electron pairs	Self-healing hydrogel, multi-responsiveness, low energy loss, and simple process	Mechanical properties, poor biological activity
	Diels–Alder reaction	Addition reaction	Thermo-responsive, mild reaction conditions, and no byproducts, high yields, and good stability	Low mechanical properties, biosafety needs further research
	Michael addition	Nucleophilic addition	Mild reaction conditions, high selectivity, high yield	Low mechanical properties, sensitive to environmental factors
	Click chemical reaction	Azide compounds, triazole compounds	Mild conditions, fast reaction rate, good selectivity, and easy purification	Low mechanical properties, sensitive to environmental factors, may trigger an immune response, or are potentially toxic
	Free-radical polymerization	Free-radical polymerization, covalent bond	High selectivity, fast reaction rate, strong controllability, simple production process, easy to serialize	Heat build-up is difficult to control, the initiator may be toxic, and there is a wide molecular weight distribution
	Enzyme crosslinking	RNA or protein	Specificity, selectivity, mild reaction conditions, good biocompatibility of products	Strongly affected by environmental factors, lower mechanical properties of the product
	Chemical crosslinker	Role of crosslinking agents	Enhanced stability and durability, improved material temperature and chemical stability, gentle, easy to handle, easy to control	Causes material shrinkage or side reactions, loss of product quality, and material calcification

#### Physical crosslinking

Ionic crosslinking involves the combination of a charged polymer with another charged polymer or multivalent ion through ionic bonding. Some natural polysaccharides and their derivatives lack high thermal stability and mechanical strength, often causing ionic crosslinking to enhance their properties [71]. Rezvanian et al. [11] prepared alginate-pectin hydrogels by ionic crosslinking, facilitating the release of simvastatin for trauma repair. Li et al. [72] designed an SA/Hardystonite (HS) hydrogel to accelerate the rate of epithelial tissue and vascular regeneration using Ca^2+^ as a crosslinking agent to promote wound healing. Ren et al. [12] obtained shape-memory hydrogels (SM) under Ca^2+^ crosslinking, wherein Ca^2+^ modification improved the mechanical strength of the gels and imparted SM to the hydrogels so that they could be restored to their initial state, allowing controlled growth at different locations. The synergistic effect of ionic crosslinking contributes to the remarkable toughening of hydrogels with no specific structural design or the addition of other reinforcing agents. This enhancement renders hydrogels capable of meeting the mechanical properties required for tissue engineering, making them more suitable for gene therapy.

Base pairing involves the interaction between two double-stranded DNA molecules via hydrogen bonds [73]. Simple base-based hydrogels have poor mechanical properties and often interact with other crosslinks. Gao et al. [74] constructed multifunctional DNA hydrogels to accelerate trauma repair. Precise base pairings between sticky ends can be constructed quickly, and cytimidine–Ag^+^–cytimidine bridging ligands complexed with Ag^+^ significantly improve the mechanical properties of hydrogels and provide slow-release, long-lasting properties outstanding for gene therapy for tissue trauma. Furthermore, the hydrogel acted on M2 macrophages through passive recruitment and release of endogenous chemokines by G-coupled protein receptors, promoting the early transition from the inflammatory phase to the proliferative phase. Tang et al. [75] used enzymatic amplification synthesis to prepare DNA strands and constructed hydrogels for tissue engineering applications using the base-pairing principle. Base pairing has excellent recognition ability and can build highly sensitive and selective biosensing and diagnostic platforms; however, its mechanical properties may be affected by environmental factors, such as temperature, pH, and ionic strength, which are detrimental to the stability of gene therapy.

A thermotropic phase transition is used to prepare hydrogels by changing the temperature of the polymer solution. The entanglement of polymer molecular chains to form a cross-linked network structure also depends on the change in solution temperature [76]. Thermotropic phase-transition-prepared hydrogels do not require chemical crosslinking agents and can be injected into the body as a liquid to achieve in situ curing. The co-existence of hydrophilic/hydrophobic groups is a key feature of thermotropic phase-transition hydrogels [77]. Wu et al. [78] prepared sEV@CS/β-GP hydrogels and used them to piggyback BMSCs-derived sEVs. These hydrogels have excellent temperature-sensitive properties, biocompatibility, and stability, with a volume that responds to changes in temperature and exosomal miR-21 that can promote vascular regeneration by targeting SPRY2. Mao et al. [79] prepared a PEG-PDLA/PLLA-PEG-PLLA hybrid hydrogel that exhibited 20 diverse thermo-responsive phase-transition behaviors suitable for the encapsulation and release of gene therapy drugs. As the thermotropic phase transition depends on temperature change, the temperature change should always be considered in hydrogel preparation, and most therapeutic genes will be degraded at an accelerated rate under the effect of high temperature. Therefore, it is crucial to consider the effect of temperature on the activity of therapeutic genes, which is necessary for the high efficiency of gene therapy.

Molecular-specific recognition involves bonding through van der Waals forces or electrostatic interactions [80]. It is highly sensitive, recognizes a wide range of molecules, and mediates biological processes essential for biochemical reactions [81]. For example, specific binding between receptors and ligands is the basis of signaling and drug action, whereas specific binding between enzymes and substrates is the basis of biocatalytic reactions [82, 83]. Hu et al. [84] prepared the amorphous CMC-ALG-EGF hydrogel by treating ALG, N-carboxymethyl chitosan (CMC), and calcium chloride with divalent chelation with epidermal growth factor (EGF) and static electricity, d. The porous structure of the hydrogels facilitates the loading and release of EGF, promoting cell proliferation and wound repair. Although the sensitivity of molecular-specific recognition is outstanding—which renders it possible to deliver therapeutic genes to damaged parts of the body with greater precision—, it binds weakly and is sensitive to environmental factors. Therefore, the preparation must be strictly controlled, and these points are worth focusing on and improving.

#### Chemical crosslinking

The Schiff base reaction is a nucleophilic addition elimination reaction that involves nitrogen atoms with lone pairs of electrons in nucleophilic reagent ammonia compounds attacking a positive charge carbonyl carbon atom, forming an α-hydroxy-amine compounds intermediate, and obtaining the Schiff base through the dehydration reaction [85]. The Schiff base reaction does not undergo a solution–gel phase transition, which makes it favorable for the filling of organ defects. Yang et al. [86] prepared HA-PEI@siRNA-29a hydrogels based on Schiff base bonds to improve the wound healing rate. Fan et al. [20] prepared CMC-oxidized chondroitin sulfate (OCS) hydrogels using the Schiff base reaction. However, the prepared hydrogels exhibited high release rates and poor mechanical properties. Therefore, they prepared BSA-loaded chitosan microspheres (CMs) using the emulsion crosslinking method and embedded them in prepared hydrogels to improve their bioactivity and mechanical properties. The survival of chondrocytes cultured in vitro significantly improved. The Schiff base reaction has excellent characteristics, such as multiple responsiveness, low energy loss, and process simplicity; however, its poor mechanical properties, poor biological activity, sensitivity to environmental factors, and biosafety deficiencies can limit its use.

The Diels–Alder (DA) reaction is a typical cycloaddition reaction used for the synthesis of cyclic and heterocyclic compounds [87]. This reaction involves the simultaneous participation of four π-electrons in the dienophile and two π-electrons in the allylic reagent, resulting in the formation of a conjugated system of six-membered rings [87]. Fujisawa et al. [88] synthesized a drug-release system using a DA reaction designed to enhance drug release in locally heated tissues. The system targets drug release, thus improving therapeutic efficiency and overcoming side effects associated with conventional drugs used in the treatment of pancreatic cancer. Li et al. [89] prepared a pectin-chitosan hydrogel using the DA reaction. The hydrogel exhibited an initial increase and a subsequent decrease in solubilization at elevated pH. The MTT assay and the cytotoxicity analysis of fibroblast L929 cells indicated that the gel showed good cytocompatibility and a favorable crosslinking density. It is effective in targeting the release of therapeutic gene drugs, enhancing the transfection efficiency of gene therapy, and is suitable for treating tissue trauma owing to the thermal responsiveness, mild reaction conditions, the absence of byproducts, and stability of the DA reaction.

Michael addition is a conjugate addition reaction that involves a nucleophilic conjugate system and a nucleophilic negative carbon ion under the catalytic action of a base. This reaction is characterized by mild conditions, strong selectivity, good stability, and high yield, making it a common method for growing carbon chains in organic synthesis [90]. Jin et al. [33] used Michael’s addition to crosslink PEG vinyl sulfone and thiolated HA in hydrogels to promote bovine chondrocyte proliferation. Tao et al. [14] prepared Gel-PDA@Cur hydrogels through the coupling of curcumin (Cur)-loaded mesoporous polydopamine (PDA@Cur) nanoparticles with PEGDA modified with chitosan by Michael addition. The gel can promote the expression of VEGF, TGF-β1, Arg I anti-inflammatory factors and inhibit the expression of TNF-α, IL-6, and CCR7 pro-inflammatory factors, as well as reduce the inflammatory response and accelerate wound healing. Furthermore, the hydrogel causes bacterial death by inducing exocytosis of K^+^, causing leakage of DNA, RNA, and proteins inside the bacteria, and disrupting the bacterial membrane. Despite the stability and high yield of the Michael addition reaction, its sensitivity to environmental factors and low mechanical properties may hinder the hydrogel from meeting tissue engineering needs, diminishing the effectiveness of gene therapy. Future research should focus on functional modification to address these shortcomings.

Click chemistry involves the Cu-catalyzed reaction of an azide with an alkynyl compound to form an enimide intermediate, which then reacts with another alkynyl compound to form a 1,2,3-triazole compound [91]. Click chemistry offers mild reaction conditions, fast reaction speed, good selectivity, and easy purification, providing additional options for the preparation of hydrogels with various specifications [92]. Ren et al. [93] prepared the glycopolypeptide by coupling 3-(4-hydroxyphenyl) propanamide (HPPA) to poly(γ-propargyl-L-glutamate) (PPLG) by click chemistry, followed by H_2_O_2_ and horseradish peroxidase (HRP) to obtain PPLG-grafted with mannose and HPPA (PPLG-g-Man/HPPA) hydrogels. Chondrocyte cultures showed strong cell viability and the content of proliferation rates, and type II collagen and GAG were significantly increased on this gel, which also showed good biocompatibility with L929 cells in vitro. However, the poor mechanical properties of chemically responsive hydrogels and their sensitivity to environmental factors (which can trigger an immune response or be potentially toxic) are detrimental to the realization of gene therapy in vivo and need to be improved and optimized.

In the presence of heat or light, precursors with unsaturated or photosensitive functional groups undergo free-radical polymerization. This method is characterized by high selectivity, fast reaction rate, modulation, and easy serialization, and is well-suited for gene therapy [94]. Zou et al. [95] prepared a Gel@Exo system with hucMSC-derived Exos that resulted in a substantial increase in retention time and a large decrease in the fibrotic area in myocardial tissues after introduction into injured rat hearts. Real-time polymerase chain reaction (RT-PCR) and immunofluorescence staining revealed that the expression of *TGF-β1*, *VEGF-A*, *VEGF-B*, *vWF*, and *Serca2a* genes, as well as myocardium-associated proteins, such as CD31, Cx43, α-SMA, and Ki67, were significantly upregulated in damaged cardiomyocytes, which had a significant therapeutic effect on myocardial infarction (MI). Free-radical polymerization is characterized by a strong modulation that can precisely control the structure and performance of hydrogels by adjusting the polymerization conditions (such as light intensity, polymerization temperature, initiator concentration, and monomer concentration), thus better adapting to different gene therapy requirements.

Enzymes are highly specific catalytic RNA or proteins produced by living cells [96]. Enzymes can significantly reduce the activation energy required for a chemical reaction or facilitate or inhibit the reaction by binding products [97]. The development of enzyme crosslinking is well established. Cai et al. [15] cross-linked MMP-2 with allyl ethylene oxide (AGE)-modified carboxymethyl chitosan (CMC) and loaded siRNA complexes targeting TGF-β1 onto it to make hydrogels. The hydrogel responded to MMP-2 upregulation in tendon tissues, resulting in the controlled release of siRNA, which reduced the negative impact on tendon healing. Furthermore, the gel has a silencing effect on the gene related to TGF-β1, which can inhibit fibroblast proliferation and differentiation, thus preventing adhesion around the tendon. The enzyme is susceptible to external factors (such as pH and temperature); therefore, it can be unstable, which requires modification of the additive material to improve its deficiencies and achieve a higher level of gene therapy effects.

Chemical crosslinkers increase the stability, hardness, strength, and durability of substances by crosslinking hydrocarbon bonds in the molecular chains [98]. Common chemical crosslinking agents include glutaraldehyde (GTA), epoxy resin, phenolic resin, isocyanate, and glutaralose. Yu et al. [99] coupled CMC with a poloxamer to form a hydrogel using GTA as a chemical crosslinker. The composite hydrogel exhibits a reversible sol–gel transition regarding the pH and temperature. Cell Counting Kit-8 experiments confirmed that the hydrogel was non-toxic to human corneal epithelial cells, confirming it as a potential material for ophthalmic drug delivery. Despite the numerous advantages of chemical crosslinkers in the materials field, there are challenges and limitations. The crosslinking process may cause shrinkage of the material or side reactions that can lead to a decrease in product quality for some specific polymer systems. Furthermore, some chemical crosslinking agents may pose non-negligible risks to humans or the environment, and these are barriers that need to be overcome.

Natural polysaccharide hydrogels have poor mechanical properties, controllability, and permeability, and are therefore not strong enough to withstand high mechanical loads, often needing to be modified with other materials. At the same time, the degradation products of some natural polysaccharide hydrogels can trigger allergic reactions, thus limiting their application. Protein hydrogels have excellent mechanical strength and biocompatibility, but their degradation behavior, bioactivity, and stability are insufficient and they are susceptible to external factors (e.g. pH, temperature). Synthetic hydrogels have good stability and maneuverability and can be well adapted to the needs of different scenarios, but their poor biocompatibility, slow degradation, and high biotoxicity need to be addressed. Researchers have tried various methods, such as introducing bioactive molecules and changing the crosslinking method, to solve these problems, but further research is still needed. Hydrogel cross-linking requires harsh external conditions, cross-linking agents, and chemical initiators, and the toxicity of chemical cross-linking has led to further discussion [6]. Future improvements of physically crosslinked hydrogels can be achieved by developing dual or multi-network structures that incorporate different types of physical cross-linking to enhance overall performance; smart material design, enhancement of the intrinsic stability and functionality of physically crosslinked hydrogels, and the realization of controllability of their performance under complex conditions. Future improvements of chemically crosslinked hydrogels should focus on smart crosslinker design, green and non-toxic synthetic routes, and enhancement of their biological activities to improve their performance [84, 86].

## Therapeutic genes

Gene therapy is closely related to therapeutic genes. RNA, microRNA, small plasmids, interfering fragments, exosomes, and aptamers are commonly used therapeutic genes, but they often suffer from easy degradation and poor stability. For instance, RNA is frequently influenced by extracellular and cellular barriers, as well as enzymes. SiRNA can activate the immune system although its cellular uptake is low. Therefore, modifications are often necessary, and zinc finger nucleases (ZFNs), transcription activator-like effector nucleases (TALENs), and CRISPR-Cas9 are commonly used modifications.

### Types of therapeutic genes

#### RNA

Ribonucleic acid (RNA) is a single-stranded molecule made up of ribonucleotides. It contains the genetic information present in cells and some viruses and virus-like organisms [100]. Transferring RNA to endoplasmic reticulum ribosomes directs protein synthesis and regulates target genes, and some RNA can be used as nucleases to catalyze intracellular reactions [101]. Although targeted RNA delivery allows disease treatment, RNA is susceptible to degradation by ribonucleases (RNases) and is hindered by extracellular and cellular barriers [102]. Therefore, researchers have attempted to encapsulate therapeutic genes in liposomes, inorganic nanomaterials, and polymeric nanoparticles (NPs) with additional hydrogel loading to improve the transfection efficiency of gene therapy [103]. The topical application of hydrogel-dispersed lyophilized TLNκ (a polymeric nanocarrier) encapsulated with locked nucleic acid (LNA) anti-miR-107 accelerates wound healing [104]. Lipid nanoparticles (LNPs) are commonly used as RNA inclusions, with outstanding gene therapy effects after additional hydrogel loading. Chitosan-SA hydrogels containing mRNA lipoplexes induce localized transfection in vivo and increase T-cell proliferation and antibody production [102]. However, liposomes are thermodynamically unstable and prone to aggregation in charged hydrogels, and published studies on RNA liposome-loaded hydrogels remain limited. Further improvement of their deficiencies is needed to develop new countermeasures for tissue trauma from gene therapy.

#### MicroRNA

MicroRNAs (miRNAs) are single-stranded RNAs encoded by endogenous genes involved in processes such as biological growth and development, differentiation, and metabolism; they also play a substantial role in gene regulation [105]. In recent years, miRNAs have been increasingly explored in tissue engineering, which has greatly expanded the value of their use [106]. Yang et al. [86] prepared composite hydrogels based on Schiff base bonds by mixing HA-ADH, siRNA-29a gene-loaded HA-polyethyleneimine (PEI) complex (HA-PEI@siRNA-29a), and oridonin (ori) loading alginate microspheres (Alg@ori) to prepare composite hydrogels. In vitro mouse fibroblast L929 experiments confirmed a good biocompatibility of the gel, which significantly shortened the inflammatory phase. In vivo experiments confirmed that it promotes the production of angiogenic factors such as CD31 and α-SMA, increasing the rate of wound healing. Pan et al. [52] used PEI-coated AuNPs coupled with miR-29b to prepare a hydrogel. Hydrogels were prepared by mixing alginate with modified gelatin with GTA and Ca^2+^, and the prepared miR-29b/AuNPs were loaded onto them. The gel scaffold can achieve bone healing; miRNA is slowly and continuously released during the process and the scaffold is gradually replaced by new tissue, which is a promising bone repair material. The miRNA hydrogel has excellent biocompatibility and natural degradation in vivo, which makes it a highly superior material; however, miRNAs are prone to mutation (since they are single-stranded fragments) which prompts a decrease in the effectiveness of gene therapy. Future studies should investigate the prevention of miRNA mutation.

#### Small plasmids

Small plasmids are independent of the cell chromosome outside the ring DNA; they exist in bacteria, archaea, and other prokaryotic organisms; carry a certain amount of genetic information; and have some autonomy and genetic characteristics through transfer, replication, and other means of transmission between cells, making them suitable carriers for genetic engineering [107]. Some small plasmids can deliver antibiotic-resistant drugs and play an important role in medicine [108]. The BMP-2 plasmid (pBMP-2) is a good osteogenic differentiation gene and promotes bone differentiation [109]. Ding et al. [110] combined chitosan microspheres (CS-MS) with hydroxyapatite lime (HAp) using an emulsification method and loaded infectious PEI/pBMP-2 onto them to prepare hydrogels. The good biosolubility and wide range of surface areas of the hydrogel allowed the sustained release of plasmids for their continuous uptake by cells adhering to the material. This system also continuously secretes relevant target proteins to promote osteogenic differentiation of bone-deficient tissues. Small plasmids are cyclic DNA molecules and are easily degraded by DNA enzymes; therefore, they often require an external carrier. The hydrogel itself is superior, and complex small plasmids can improve the efficiency and stability of gene transfection and better achieve gene therapy for tissue trauma.

#### Interference fragments

Interference fragments mainly refer to siRNAs, which can silence almost any target gene after entering the cell, and are important for gene regulation [111]. siRNA specifically cleaves and degrades mRNA with the complementary sequence of a gene substrate, targeting a specific mRNA to inhibit the expression of target genes when linked to the RNA-induced complex (RISC) [112, 113]. RNA interference (RNAi) is a process involving short double-stranded RNAs (dsRNAs) that leads to sequence-specific degradation. During RNAi degradation, the imported strand is recognized and binds to the complementary sequence of the target mRNA, resulting in mRNA degradation, affecting gene expression and regulatory functions [111]. Tang et al. [66] synthesized the SeMSN(siCFL1)@ P(MNs)Ang2 complex and used it to initiate RNAi under X-ray irradiation to inhibit radiotherapy-resistant glioblastoma (rrGBM) invasion in situ (Fig. 2B). Wang et al. [114] prepared a siRNA/NP complex and embedded it in a PEGb-PLA-b-DM hydrogel, which silenced Wwp1 expression and accelerated fracture repair. However, interfering fragments activate the immune system and exhibit instability and low cellular uptake, thus posing challenges for practical applications.

#### Exosomes

Exosomes are small membrane vesicles produced by cells and released outside the cell; they contain RNA, lipids, and proteins, are widely considered key carriers of information transfer between cells, and are widely used in tissue engineering [115]. Human mesenchymal stem cell-derived Exos slow degeneration and seal annulus fibrosus damage during disk degeneration [86]. Kuang et al. [116] prepared Exo@miR-26a with microRNA-26a (miR-26a) and BMSC-sourced Exos and piggybacked it onto a hydrogel. The hydrogel had a significant inhibitory effect on osteoblast differentiation genes, achieved miR-26a-targeted release, and demonstrated an efficient ability to induce osteogenic differentiation in a rat model with cranial bone defect. Yu et al. [117] isolated antagomiR-708-5p-loaded exosomes from patient plasma and loaded them onto HA hydrogels to treat femoral fracture nonunion in mice. Compared with the blank group, the Exos-equipped HA hydrogel was much more antibacterial, anti-inflammatory, biocompatible, and mechanically supportive, and significantly promoted bone differentiation and accelerated fracture healing. Although Exos have a cellular messenger function and can participate in cellular communication, they are not specific enough for target cells and may have toxic effects on non-targeted cells; thus, further research and optimization are needed to improve these deficiencies.

#### Aptamer

Aptamers are single-stranded oligonucleotides screened in vitro that can fold into a defined structure and bind to a target protein, inhibiting protein–protein interactions for disease treatment [118]. Aptamers are easy to screen and chemically modify, have low-ionic-strength dependence, exhibit good stability in vivo and in vitro, and can serve as drug delivery carriers to precisely deliver drugs into cells, such as chemotherapeutic agents, therapeutic RNAs, and radioisotopes. Systematic evolution of ligands by exponential enrichment (SELEX) is a common method for the large-scale in vitro synthesis of aptamers [119]. Mesenchymal stem cell-specific aptamers enable gene therapy in osteoporotic bone regeneration [120]. Liang et al. [121] used an osteosarcoma (OS) cell-specific aptamer (LC09) to promote the selective distribution of in situ OS and inhibit the effects of in situ OS malignancy and metastasis, which significantly increased bone mineral density in osteoporotic mice. The aptamer is unstable in vivo, has a short half-life, and is prone to degradation, which can compromise the effectiveness of gene therapy. The aptamer can be modified by chemical or genetic modification to improve its stability, biocompatibility, and function in the field of gene therapy tissue engineering.

### Modifying therapeutic genes

**Fig. 3:**
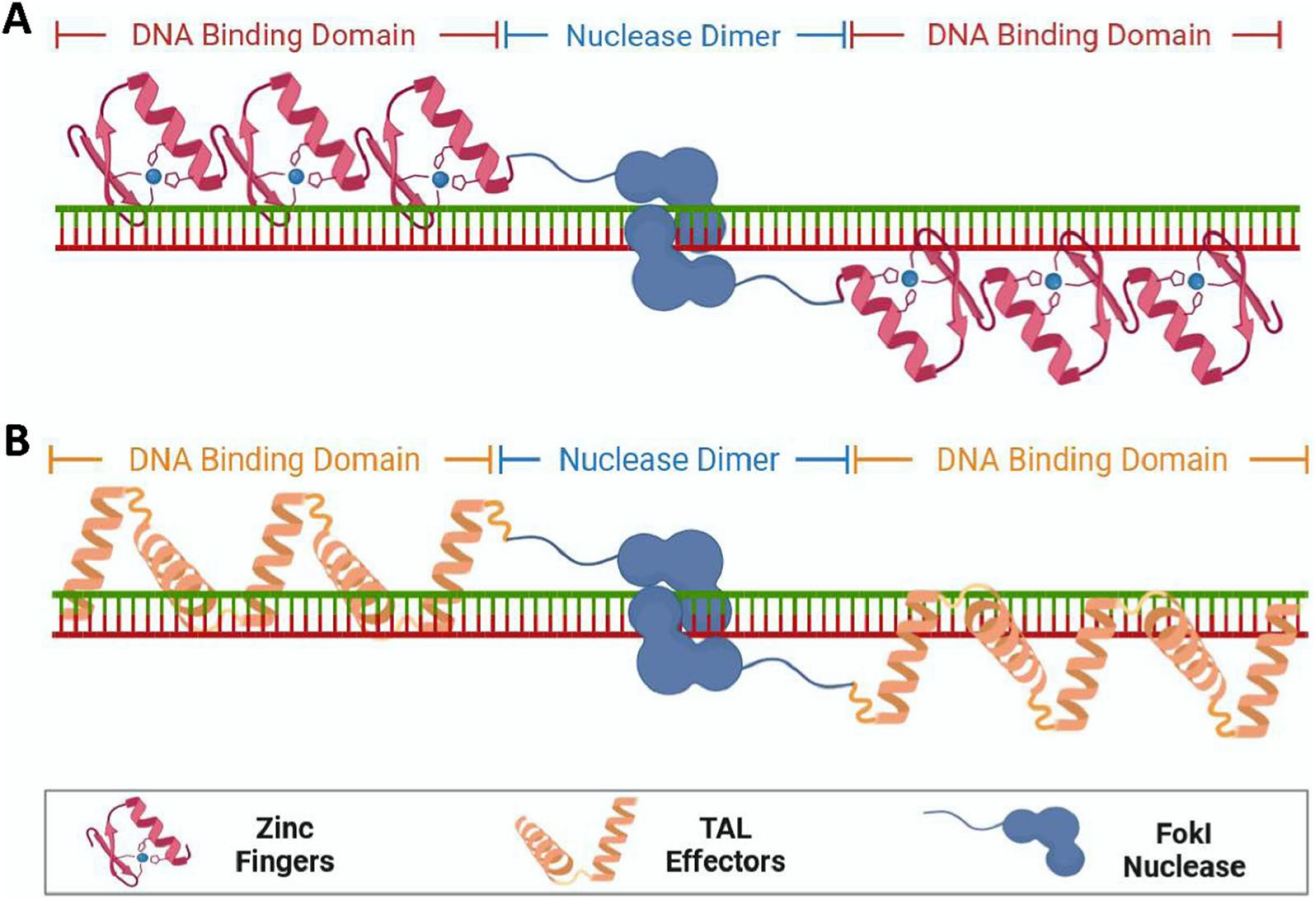
Mechanism of the action of therapeutic gene modification modality. **A** ZFNs. **B** TALENs.  Created with BioRender.com. (Agreement number: LO26NU7V24)

Gene therapy is inseparable from processing and modification, and ZFNs, TALENs, and CRISPR-Cas9 are commonly used modifications [122]. TALENs and ZFNs rely on DNA interactions to edit genomic sites, and CRISPR-Cas is designed to achieve specific localization of genomic DNA sequences through complementary RNA strands [123]. ZFNs are artificially constructed proteins comprising several zinc finger structural domains fused to a type IIS restriction endonuclease FokI, which are used for specific DNA triplexes, DNA sequence recognition, and editing of targeted genomes (Fig. 3A). However, the ZFN structural domains suffer from limited modularity, lack of specificity, and off-target cleavage, which results in poor performance [124]. TALENs avoid these limitations by significantly increasing the amount of chromosomal double-strand breaks at the locus and joining the broken double-strand to form the desired genetic modification through homologous recombination (HR) and non-homologous end joining (NHEJ) (Fig. 3B); thus, they exhibit a higher editing efficiency compared to ZFNs [125]. TALENs act as a pair of “DNA scissors” that can precisely edit genomic loci and were widely used in the genetic studies of most species [126]. Chen et al. [127] designed TALENs that are applicable to DNA-conserved regions of different genotypes of hepatitis B virus (HBV) to promote the transcription of HBV inhibitor interferon (IFN)-stimulated responsive elements. The synergistic effect of IFN-α opens a new avenue for gene therapy of chronic hepatitis B (CHB).

**Fig. 4:**
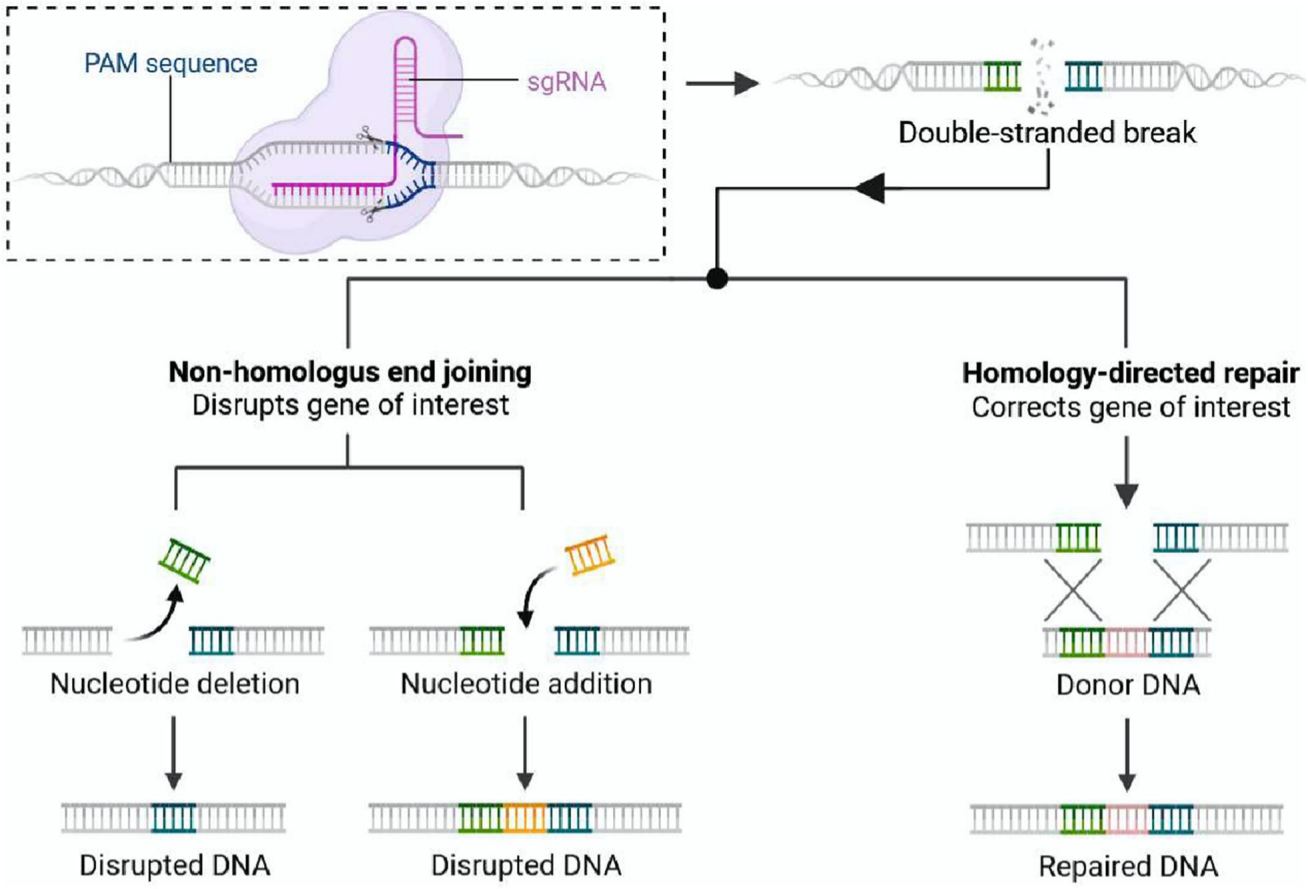
Principles of CRISPR-Cas9 gene editing technology. Created with BioRender.com. (Agreement number: ZT26NU7MMY)

Although TALENs are already an excellent gene modification technology, they are limited by the complexity of the preparation process, the large production cost, and the high number of amino acids involved [125]. Thus, the more versatile CRISPR-Cas9 has become the new generation of “DNA scissors” [128, 129]. CRISPR, known as “Clustered Regularly Interspaced Short Palindromic Repeats,” is a specialized sequence found in bacteria and some archaea that plays an active role in defending the immune system against invasion by exogenous substances (such as phages) [130]. Cas9 refers to “CRISPR-associated protein 9,” an enzyme used to cut specific DNA sequences. Fujikura et al. [131] added a medium-chain triglyceride-containing ketogenic diet (MCTKD) to promote skeletal muscle regeneration in CRISPR-Cas9 gene-edited Duchenne muscular dystrophy rats. Furthermore, Freitas et al. [132] gene-edited MSCs with CRISPR-Cas9 to enhance gene expression of genes related to the BMP/TGF-β signaling pathway to overexpress BMP-9. CRISPR-Cas9 is a powerful genomic editing technology widely recognized for its broad range of applications, precise gene editing, and low cost (Fig. 4). However, this method has limitations such as immunotoxicity, low HR efficiency, PAM limitations, off-target effects, and differences in editing efficiency. Therefore, it is worthwhile to further improve it for therapeutic gene modification.

The development of therapeutic genes has brought great hope for treating human diseases; however, they usually exhibit problems such as easy degradation, instability, mutability, and safety, and some may trigger immune responses, interference with healthy cells (such as neuronal cell damage or death), and the risk of tumors. Although ZFNs, TALENs, and CRISPR-Cas9 modifications are widely used in gene therapy, they have shortcomings. The activity of ZFNs is affected by the ability of zinc finger proteins to bind to DNA, as well as by factors such as the DNA sequence and the intracellular environment, rendering their action unreliable and limited. Although TALENs and CRISPR-Cas9 are characterized by high efficiency and precision, the construction and design of TALENs require customized modifications for different genomes, which are costly and suffer from immune responses and off-target effects. CRISPR-Cas9 still has limitations, such as off-target effects, immune responses, and gene polymorphisms. Therefore, future research should focus on improving the deficiencies of therapeutic genes and their modification.

## Loading of therapeutic genes into hydrogels

Therapeutic genes are often affected by cellular barriers, enzyme sensitivity, and difficulty in transport to cellular compartments; therefore, carrier loading of therapeutic genes is necessary. Physical and chemical loadings are commonly used strategies; the former is stable, controllable, and free from the addition of chemical reagents, manifesting low or no toxicity. The chemical loading varies in toxicity owing to the addition of chemical reagents with flexible adjustability, which has attracted the interest of researchers. In the following sections, we organized the loading methods, principles, and characteristics of gene therapy (Table 3).

**Table 3:** Loading method of therapeutic genes

Types	Loading method	Principle	Loaded gene	Advantages	Ref
Physical loading	Freeze-drying	Freezing, lyophilization	RNA	High stability	[133, 134]
	Injection	High-pressure gas injection	siRNA	Low pollution, simple process	[135, 136]
	Centrifugal coprecipitation	Mixing of the original gel solution, centrifugation, precipitation of the gel	mRNA	High precision, simple process	[137, 138]
Chemical loading	Hydrogen bond	Electrostatic interaction forces between atoms	siRNA	Mechanical properties, reversibility	[139, 140]
	Ionic bond	Electric charge interaction	mRNA, siRNA	Antimicrobial, frost resistance, and electrochemical stability	[12, 141]
	Covalent bond	Common electron pair	siRNA	Good elasticity, self-healing	[43, 142, 143]
	Hydrophobic interaction	Pro/hydrophobic attraction or repulsion	siRNA	Highly sensitive, reversible	[102, 144]

### Physical loading

#### Freeze-drying method

Freeze drying is a widely used method of gene loading that involves mixing of solvents and polymers, followed by freezing and lyophilization to force a pressure drop, which removes the water in the solution that drives the mixture to sublime, resulting in the formation of an interconnected porous structure [133]. During freeze drying, therapeutic genes are encapsulated in the hydrogel skeleton, thus avoiding the problem of gene activity being easily damaged by normal drying methods. Simultaneously, low-temperature drying conditions can reduce the possibility of gene mutations and maintain biological activity [134]. Fiorica et al. [145] obtained HA-EDA-g-α-elastin/HA hydrogels by freeze drying, which were outstanding mechanically and rheologically and compensated for the poor adhesion of HA cells. The sustained release of VEGF was effectively controlled in endothelial cell cultures to promote adsorption, proliferation, growth, and induction of tubular structure formation in human vascular endothelial cells (HUVECs), which are suitable for vascular neovascularization and soft tissue regeneration. Although freeze drying can reduce the mutation rate, the therapeutic genes in the hydrogel are encapsulated in a solid skeleton and pores or cracks may form after freeze drying, which can lead to poor release of therapeutic genes.

#### Injection method

The injection method is a loading method wherein genes are introduced into a hydrogel using high-pressure gas jet injection [135]. This injection method can evenly disperse therapeutic genes in the backbone of the hydrogel in a short time, improving the loading efficiency and reducing the loss of activity by avoiding direct contact with the external environment. Spraying siRNA-containing hydrogels onto arthritic mice specifically silenced genes related to the pathogenesis of rheumatoid arthritis (RA) and achieved an anti-RA induction effect [29]. Wang et al. [136] introduced a hydrogel containing siRNA against MMP-2 (siMMP2) in a mouse model of MI by injection. The hydrogel was eroded by proteases, resulting in the release of active siRNAs, which reduced the MMP2 content of cardiac fibroblasts and improved myocardial thickness in MI. Moreover, the hydrogel improved myocardial blood output, ejection fraction, and cardiac output in the MI region. The spraying process may lead to damage or deformation of the hydrogel structure since it requires the use of high-pressure or high-speed airflow, thus affecting its stability and release performance and reducing the effect of gene therapy.

#### Centrifugal coprecipitation method

Centrifugal coprecipitation enables efficient loading of therapeutic genes. Centrifugation allows the coprecipitation of therapeutic genes with hydrogel particles, thus achieving uniform distribution in the hydrogel. This method can improve the efficiency of gene loading and stability of genes, which is conducive to improving therapeutic effects [137]. The advantages of the centrifugal coprecipitation method are as follows: the loading process is simple, does not require complex equipment and operations, does not require the addition of chemical reagents, and has a relatively low impact on the integrity and activity of therapeutic genes. Furthermore, the centrifugal coprecipitation method loads gene therapy hydrogels with a denser structure, a higher degree of crosslinking, and greater controllability, which helps to realize the sustained release of therapeutic genes [138, 146]. However, centrifugation can lead to structural changes in the hydrogel that affect its performance and release behavior. Therefore, the properties and structure of the hydrogel should be considered, optimized, and adjusted accordingly when centrifugal coprecipitation is used.

### Chemical loading

#### Hydrogen bonds

A hydrogen bond is an electrostatic interaction between H^+^ and an extremely electronegative atom (typically nitrogen, oxygen, or fluorine) [139]. Hydrogen bonds are important for stabilizing the structure between molecules, catalyzing chemical reactions, aiding the intermolecular transfer of substances, and changing melting and boiling points [147]. For example, the rate of in vitro release of siRNA from HA-poly(vinyl alcohol) hydrogels is slower than that from poly (vinyl alcohol) hydrogels owing to the presence of more hydrogen bonds between the HA backbone and siRNAs [148]. Chen et al. [140] prepared siRNA@G5-PBA@Gel hydrogels by modifying therapeutic siRNA to silence the expression of P65 with G5-PBA under the effect of hydrogen bonding, which had a slow release of siRNA in vitro/in vivo and an inhibitory effect on inflammatory factors and is suitable for precision therapy of intervertebral disc (IVD) generation (IVDD). The different materials of the gene therapy hydrogel indicate that the material and molecular weight may affect the ability and stability of hydrogen bond formation and that the gene release behavior of the hydrogen-bonded hydrogel may be affected by environmental factors, leading to further destabilization of the hydrogen bond that can affect the efficacy of the final tissue engineering application.

#### Ionic bonds

Ionic bonds are chemical bonds formed by charge interactions between metal and nonmetal ions and have high melting and boiling points [149]. Ren et al. [12] prepared SM hydrogels by crosslinking with Ca^2+^ to compensate for this deficiency. Li et al. [40] demonstrated that an ionic product synergized with a small interfering RNA for MMP-9 (MMP-9-siRNA) improved the synthesis of the ECM protein and decreased the expression of MMP-9 in cells. Chitosan particles loaded with MMP-9-siRNA in 45S5 Bioglass®/SA hydrogels significantly reduced MMP-9 expression, promoted collagen synthesis, and accelerated vascular regeneration and wound healing rate, showing great potential in diabetic wounds. However, the gene release of ionic bonding can be affected by environmental factors such as pH, ionic strength, and temperature, and a small portion of the ionic bonding process can produce harmful byproducts that can have toxic effects on cells; the ionic bonding of the hydrogel lacks resilience and mechanical strength, which can affect the effectiveness of gene therapy.

#### Covalent bonds

Covalent bonds form stable structures by sharing one or more electron pairs to fill the valence electron layers [150]. Cai et al. [43] prepared nanoparticles with siRNA and modified them to prepare GelMA MS. Subsequently, HA-ADH and HA-CHO were coupled and used to modify siRNA@MS using the hydrazone bond as a linkage to obtain the siRNA@MS @HA hydrogel, which showed good elasticity, obvious self-repair in the rheology test, and release of on-demand siRNA, which was effective in preventing tendon sheath adhesion in the damaged part of the rat tendon. Covalent bonds are more stable; however, the release of therapeutic genes in the covalent bond is usually irreversible, which limits the effectiveness and durability of gene therapy, contrary to the sustained slow release required for traumas such as bone defects, rendering it difficult to achieve good therapeutic effects.

#### Hydrophobic interactions

Hydrophobic interactions occur when a substance is in contact with water and repels and prevents water molecules from entering its interior. Substances with similar hydrophobic properties attract each other, whereas those with different properties repel each other [151]. Hydrophobic interactions mediate the self-assembly of succinylbutanediol nanomaterials to prevent breast cancer metastasis by inhibiting the expression of VCAM-1 [144]. Kim et al. [152] prepared polyethyleneimine-poly(organophosphoronitrile) conjugates coupled with siRNA to form composites and transformed them through hydrophobic interactions into complex hydrogels, which induced slow release of polyplexes through solubilization and degradation and achieved antitumor effects through cell cycle protein B1 gene silencing. However, the ratio of the hydrophobic to hydrophilic regions of such hydrogels must be strictly adjusted to maintain the stability of their hydrophobicity, thus making the loading modification of gene therapy hydrogels extremely stringent.

The loading of therapeutic genes into hydrogels has made progress, but it still faces challenges. Physical loading is a notable approach owing to its relative simplicity of operation, biocompatibility, and controllability; however, it suffers from shortcomings such as low loading efficiency and difficulty in controlling release behavior. The physical loading of hydrogels may lead to gene aggregation or precipitation, which is insufficient for efficient gene therapy. Although chemical loading is characterized by controllability and high loading efficiency, its immunogenicity, cytotoxicity, and stability still do not meet the requirements; chemical loading can inactivate and denature, and the stability and durability of chemical bonding cannot meet the demand. Smart-responsive cross-linking agents, cell-affinity modifications, development of novel non-toxic, low-immunogenic materials, and combining gene therapy with other therapeutic modalities (photothermal therapy, immunomodulation, etc.) should be the key strategies for future research on loading therapeutic genes into hydrogels.

## Characterization of gene-loaded hydrogels

To enhance therapeutic gene protection, improve delivery efficiency, and ensure transfection efficiency, detailed characterization of loaded gene hydrogels is essential. Parameters such as the aperture size, mechanical properties, and transfection efficiency of hydrogels need to be evaluated.

### Aperture size

Aperture size is a key parameter for loading gene hydrogels and is an important indicator of the hydrogel pore structure, loading capacity, and gene release rate. A large aperture size provides more space for the loading of therapeutic genes but leads to an excessively fast release, whereas a small aperture limits the amount of gene loading and the release rate [153]. The aperture size can be measured using the nitrogen adsorption–desorption technique, the pressed mercury method, and the aperture size analyzer [102, 154]. Gene therapy hydrogels are commonly desired for slow release in tissue engineering applications that include cartilage repair, spinal cord repair, and tumor therapy; therefore, the aperture size is more demanding. In cardiac repair, the aperture size of the hydrogel should be appropriate to avoid being too large, which results in blood vessel blockage. Similarly, eye treatment also requires an appropriate selection of aperture sizes. Therefore, the aperture size should be planned according to the expected loading and release requirements when designing gene carriers. The design and regulation of aperture size must be further optimized based on a more comprehensive understanding of its mechanisms of action to achieve more efficient, safe, and precise gene therapy [153].

### Mechanical properties

Mechanical properties refer to the mechanical properties of a material under force, including strength, toughness, hardness, and ductility. The mechanical properties of hydrogels play prominent roles in cell proliferation, differentiation, and organ recovery. Hydrogels often have poor mechanical properties; thus, they are difficult to meet the requirements of tissue compression. Therefore, researchers have made significant efforts to improve these properties of hydrogels [13]. The mechanical properties of hydrogels can help researchers assess how stable and deforming they become. A high level of stiffness and stability provides better results for the encapsulation and protection of genes and ensures the stability and continuity of gene delivery. Bone-regeneration hydrogels with excellent mechanical properties can effectively support damaged bones. The mechanical properties of gene-loaded hydrogels are significant factors that affect their use as gene carriers. The hydrogel mechanical properties and preparation process need to be further optimized to provide more suitable and stable carriers for gene therapy.

### Biocompatibility

Biocompatibility refers to the ability of a material to co-exist with tissue cells in vivo without causing significant immune or inflammatory reactions. Good biocompatibility is a prerequisite for efficient gene delivery and tissue repair in gene therapy hydrogels. In gene therapy, commonly used hydrogel materials (including natural macromolecules such as collagen and HA and synthetic macromolecules such as polyvinyl alcohol and polyacrylamide) have good biocompatibility and can interact with tissue cells and promote cell adhesion and differentiation [18]. While biocompatibility primarily concerns the interactions between the material and the organism, safety is further refined to the potential risks or side effects that may result from these interactions. Therefore, at the level of material safety, gene-loaded hydrogels must undergo an exhaustive toxicological evaluation. The loaded genes must also be rigorously screened and validated to ensure that they do not contain harmful sequences and to avoid adverse reactions [155]. Therefore, the biocompatibility of gene-loaded hydrogels influences their applications in tissue engineering.

### Degradability

**Fig. 5:**
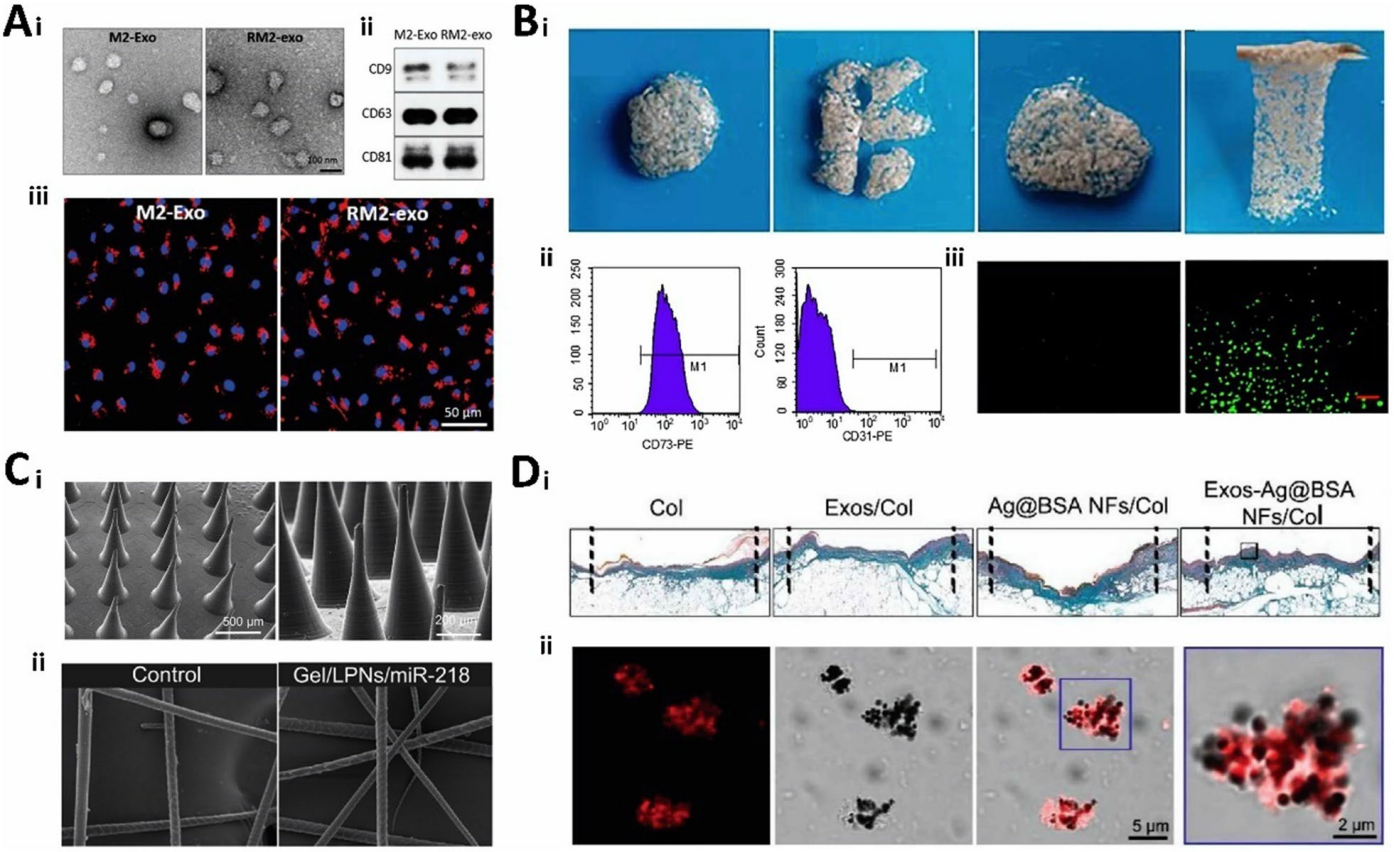
Characterization of gene therapy hydrogels. **A** Characterization of 8P-TS Exogel. (i) Transmission electron microscopy images; (ii) Expression of exosomal tetraspanin; (iii) Cellular uptake of M2-Exos or RM2-Exos. (Reproduced with permission from Ref. [64], Copyright 2022, John Wiley and Sons). **B** Characterization of the CHA/SF/GCS/DF-PEG hydrogel. (i) Self-healing of hydrogels; (ii) Flow cytometry analysis; (iii) LIVE/DEAD staining. (Reproduced with permission from Ref. [65], Copyright 2020, Frontiers). **C** Scanning electron microscopy image. (i) Scanning electron microscopy image of microsphere hydrogels; (ii) SEM image of hair after several days of microsphere hydrogel treatment. (Reproduced with permission from Ref. [156], Copyright 2022, Royal Society of Chemistry). **D** Correlation characterization of Exos-Ag@BSA NFs/Col hydrogels. (i) Masson’s trichrome staining; (ii) Exos loaded on Ag@BSA NFs. (Reproduced with permission from Ref. [31], Copyright 2023, John Wiley and Sons)

Degradability refers to the ability of a material to break down and be excreted from the internal environment of the body, thus ensuring that the hydrogel does not produce toxic and inflammatory reactions in the human body. It directly affects the rate of hydrogel decomposition; therefore, it is important to control gene release and maintain its duration. Bone-regeneration hydrogels should not degrade too quickly and should have adequate mechanical support and excellent mechanical properties [115]. Researchers have designed degradable hydrogels to piggyback exosomes to maximize the efficacy of skin wound healing (Fig. 5A) [64]. In wound repair, hydrogel degradation maximizes the conditions for vascular infiltration and provides advantages for painless gel removal; therefore, good degradability is necessary. The rate of degradation of gene therapy hydrogels is determined by the type of material, the crosslinking method, and tissue coordination. Meanwhile, the degradability required for tissue repair also varies, which undoubtedly highlights the flexible, customizable, and controllable advantages of synthetic hydrogels. Degradation products of hydrogels must be harmless to the human body from the point of view of degradability. Moreover, the speed of degradation rate is also critical. Degradation that is too fast may result in the loaded genes not functioning adequately; degradation that is too slow may trigger long-term inflammatory or foreign body reactions. Therefore, long-term safety assessment is necessary. Animal and clinical trials can help visualize the degradation of hydrogels in vivo, the effect of degradation products on surrounding tissues, and possible immune responses.

### Mechanism of hydrogels

Hydrogels can be cross-linked by physical or chemical means, as described in Sect. “Crosslinking of hydrogels”. Physically cross-linked hydrogels can be formed through interactions, but they usually exhibit instability and reversible crosslinking; they also cause morphological and structural changes when subjected to external influences (such as temperature, pH, oxidative stress, light, and ionic strength). Therefore, they are mainly used in self-healing hydrogels and responsive hydrogels, which are also useful in bone regeneration, skin trauma, and tumor therapy [157]. Chemically cross-linked hydrogels exhibit excellent stability and mechanical properties, and the mechanism of the gel can be adjusted by the addition of chemical crosslinking agents during the gelation process. However, this can reduce gel water absorption and cause toxicity to the realization of therapeutic genes, which cannot be ignored before the applications of chemical cross-linked hydrogels in tissue engineering [10]. Physical and chemical crosslinking has its advantages and disadvantages; however, they also provide a variety of options for different areas of tissue engineering.

### Transfection efficiency

Transfection is the introduction of an exogenous gene into a cell, and transfection efficiency is a measure of the degree of success of the introduction of an exogenous gene during transfection. Transfection efficiency directly affects gene delivery and expression. Methods used to assess transfection efficiency include fluorescence staining, PCR analysis, western blotting, and flow cytometry [158, 159]. RNA is highly susceptible to degradation by RNase in vitro, and there is a problem of low transfection efficiency when it is directly mixed with the hydrogel. Therefore, researchers encapsulated therapeutic genes in liposomes and polymeric NP vectors and loaded them onto a hydrogel to improve their transfection efficiency (Fig. 5C) [156]. Hydrogel microspheres (MSs) have attracted considerable attention in the field of minimally invasive tissue repair, and amino acid coupling is a popular method for enhancing gene transfection efficiency [160]. The high transfection potency of the complexes prepared for the release of miR-140 nanoparticles compensated for the short half-life, low transfection rate, and susceptibility to inactivation caused by direct injection of crude miRNAs [161]. During hydrogel transfection, the transfection efficiency is affected by numerous factors, including the composition, structure, and property of the hydrogel and the type and state of the introduced recipient cells, which affect the final effect of gene therapy.

### Safety

The safety assurance of gene therapy hydrogels requires comprehensive consideration in several dimensions. In terms of material selection, preference should be given to hydrogel materials with superior biocompatibility and low immunogenicity, aiming to minimize potential irritation and immune response to the body. At the same time, the degradation rate of the material needs to be compatible with the natural process of wound healing to ensure that it does not adversely affect the neoplastic tissue. Secondly, optimization of the preparation process is also a key step in ensuring safety. By improving the preparation technique, the impurities and residues in the hydrogel can be effectively reduced, and its purity and stability can be enhanced [6]. Furthermore, the design of gene carriers (e.g., hydrogels) should also take safety and efficacy into full consideration. The use of vectors with specific targeting can accurately deliver genes to target cells and avoid interference with non-target cells. At the same time, factors such as toxicity, immunogenicity, and stability of the vector should also be fully evaluated to ensure its safety in vivo. Moreover, a personalized treatment plan should be developed according to the patient’s specific condition and wound healing needs. In the selection of genes, the use of genes with well-proven efficacy and high safety should be favored, and the regulatory mechanism of gene expression should be taken into account to prevent the possible risk of gene overexpression or abnormal expression. Finally, gene therapy hydrogels must be thoroughly evaluated and tested before clinical application. This includes, but is not limited to, an in-depth evaluation of its biocompatibility, degradation rate, gene transfection efficiency, drug release kinetics, and other properties, as well as a rigorous prediction and assessment of potential risks and side effects [54]. These efforts will help reduce potential risks and enhance the safety and efficacy of gene therapy hydrogels in tissue engineering repair, leading to safer and more effective treatment options for patients [155].

### Other characterizations

Hydrogel chargeability affects the loading and release of genes, and the adsorption and release properties affect the loading and release efficiency of genes. In tumor therapy, excessive hydrogel crosslinking hinders the release of RNA and reduces the therapeutic efficiency. Hydrogels with controlled crosslinking can be released on demand for gene therapy and continue to act on target tissues [161]. The attractiveness of the bone tissue hydrogel should be sufficient to mimic high-water content ECM to provide the environment needed for bioactive molecules or damaged cells. Controlled drug release should also be noted. Hydrogels used in trauma repair should be safe, anti-infectious, and appropriately dosed to ensure safe and effective therapeutic results (Fig. 5D) [32]. Gene therapy hydrogels have great potential for cartilage repair, although careful consideration should be given to the type of gene, the delivery system, the tissue engineering scaffold, safety, efficacy, and durability before use. In spinal cord therapy, attention should be paid to gene type, delivery system, treatment time, dosage, long-term efficacy, and monitoring. To achieve gene therapy more efficiently, it is important to understand and evaluate its various characteristics to guide tissue repair. The demands for different hydrogels with different physicochemical properties and characterization parameters are also different and worth discussing.

The characterizations of the gene-loaded hydrogel affect its use as a gene carrier, and the physicochemical properties of the hydrogel can be understood to optimize its preparation process and performance through characterization tests. The biocompatibility and safety of the gene therapy hydrogel in cells and organisms can be evaluated using this method. Gene therapy hydrogels should be designed and applied with a greater focus on the need for personalized treatment. Different diseases and different patients have different gene expression and treatment needs. Therefore, hydrogels with specific gene drug and release properties should be tailored to achieve personalized and precise treatment. In order to achieve this goal, new hydrogel materials with excellent biocompatibility, stability, and controlled degradation should be developed; gene drugs with high efficiency, low toxicity, and high specificity should be screened; and the structure and properties of hydrogels should be modulated to achieve precise drug release and targeting.

## Hydrogel designs to enable therapeutic gene release

The release of therapeutic genes cannot be achieved without the design of the carrier. Hydrogels are a class of materials suitable for this purpose. The design of smart-responsive hydrogels is a priority because these hydrogels respond to changes in temperature, pH, light, and other factors. Furthermore, nanomaterials/microspheres loaded with genes and combined with hydrogels are a key design strategy that can increase the carrying capacity of the genes, protect the genes, and slow the release of the genes to better realize gene therapy for damaged tissues.

### Responsive hydrogels

#### Temperature-responsive hydrogels

Temperature-responsive hydrogels were extensively studied for piggybacking and the release of therapeutic genes, which do not require additional copolymerization agents or chemicals [162]. Hong et al. [163] prepared temperature-responsive hydrogels with imidazole-poly(organophosphazenes) that interact with macrophages and histamine receptors to produce MMP-9, remodel the fibrotic ECM, and reduce the bridging effect. Liu et al. [164] reported that miR-130a secretion modulates BMSC-sEVs, which stimulate the PTEN/AKT signaling pathway to promote bone regeneration. Wu et al. [78] prepared a temperature-responsive hydrogel with β-glycerophosphate-modified chitosan to rely on BMSC-derived sEVs, which had excellent temperature-sensitive properties, biocompatibility, and stability, and the BMSC-derived miR-21 exosome miR-21 promoted vascular regeneration by targeting SPRY2.

#### pH-responsive hydrogels

pH-responsive hydrogels comprise cross-linked polymers containing specific functional groups that absorb excess water under acidic conditions, resulting in volume expansion [22]. pH-responsive hydrogels can be created by atom transfer radical polymerization (ATRP). pH-responsive carboxymethylcellulose hydrogels activate the VEGF signaling pathway by loading plasma exosomes, leading to wound repair in type 1 diabetic mice [165]. Khaled et al. [166] reported a pH-responsive hydrogel on which siRNA loading could be used to slow the release of siRNA by cation-adjusting the pH of the hydrogel. The siRNA was safeguarded against enzymatic degradation and in vivo microenvironmental influences. After intravenous administration, CXCR4 siRNA was delivered to the cytoplasm of breast cancer cells to silence the expression of the CXCR4 protein.

#### Hydrogels responsive to oxidative stress

Oxidative stress-responsive hydrogels typically comprise polymers containing oxidation-sensitive functional groups that react with oxides, resulting in changes in the volume and structure of the hydrogel and responsive behavior by releasing specific substances. In gene therapy hydrogels, oxidative stress responsiveness allows site-specific targeting of genes to treat tissue damage [40]. For example, atopic dermatitis can be treated by modulating oxidative stress [167]. Lei et al. [168] prepared a biodegradable tannic acid-siRNA nanohydrogel based on environmental reactive oxygen species (ROS) stress, the release of therapeutic agents into cells to silence the expression of the *MMP-9* gene, synergistically advancing macrophage polarization and vascular regeneration to reduce the degree of chronic inflammation in wounds and promote wound healing.

#### Pressure-responsive hydrogels

Pressure-responsive hydrogels that respond to changes in external pressure typically comprise cross-linked polymer networks containing polymer chains that slide over each other. Hydrogels that respond to pressure contain a drug-containing substance released after pressure stimulation [169]. Zhao et al. [170] screened siRNA that effectively interferes with TGFβ1 expression and used it to prepare an siRNA-TGFβ1-337 transdermal patch, which is a combination of siRNA-TGFβ1-337 and pressure-responsive adhesive hydrogel. It downregulates the expression of type I collagen, promotes scar fibroblasts, and reduces scar size. In conclusion, pressure-responsive hydrogels are smart materials with important applications; however, their properties and applications require further exploration.

#### Photo-responsive hydrogels

Photo-responsive hydrogels comprise polymeric materials (polyvinyl alcohol, acrylamide, and amphoteric polymers). When the material is exposed to specific wavelengths, its crosslinks break, leading to gel dissolution [171]. Photo-responsive hydrogels are suitable for cell culture and controlled release of therapeutic genes and can be used for tissue engineering therapies [26]. Huynh et al. [171] reported a light-responsive hydrogel for on-demand delivery of genes that modulated siRNA release without impairing the biological activity upon exposure to UV light. Yang et al. [172] designed a photo-responsive hydrogel based on a photoinduced imine crosslinking (PIC) reaction. Under light irradiation, an antiadhesion barrier attached to the tissue surface was formed, and L929 cells showed excellent cytocompatibility. Light-responsive hydrogels have important applications in tissue engineering; however, excessive UV irradiation can lead to mutations in gene therapy drugs, reducing the therapeutic efficacy.

#### Ionic-strength responsive hydrogels

Based on the interaction of specific functional groups on the polymer chain with ions in solution, the functional groups adsorb or desorb ions when the concentration of ions in the environment changes, thus causing changes in the gel volume and structural rearrangements. Zhou et al. [10] prepared SA-polyacrylamide hydrogels by crosslinking them with different concentrations of Cu^2+^, Ca^2+^, and Zn^2+^. This has substantially improved the antimicrobial and mechanical strength of the hydrogel. The in vitro molecular biology assay showed good expression of VEGF and TGF-β, and the compatibility in *Escherichia coli* was outstanding, which is suitable for trauma repair. Li et al. [72] designed an SA/HS hydrogel to accelerate the rate of epithelial tissue and vascular regeneration for wound healing using Ca^2+^ as a crosslinking agent. However, there are few specific applications for ionic-strength-responsive hydrogels, and their specific properties and potential applications require further investigation.

### Nanomaterials loaded with genes and combined with hydrogels

**Fig. 6:**
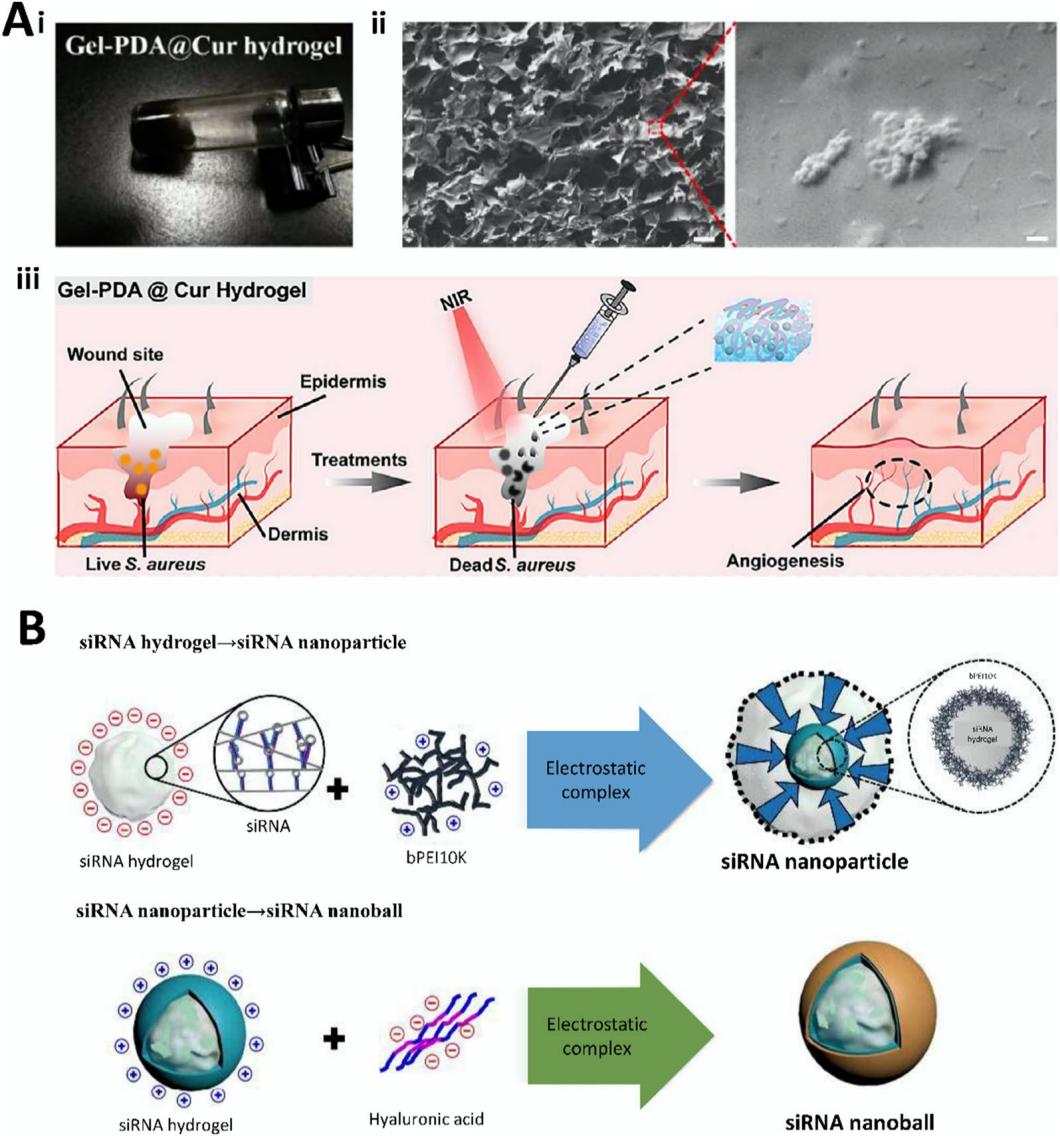
Hydrogel design for therapeutic gene release. **A** Nanomaterials loaded with genes and combined with hydrogels. (Reproduced with permission from Ref. [14], Copyright 2021, Elsevier). **B** Microspheres loaded with genes and combined with hydrogels. (Reproduced with permission from Ref. [177], Copyright 2017, Royal Society of Chemistry)

Nanomaterials typically range in size from 1 to 100 nm, and the biological properties of the material significantly change (Fig. 6A) [14]. RNA loading into nanomaterials combined with hydrogels can improve the retention time around tumors and the uptake of RNA into cancer cells [173]. Qin et al. [174] prepared miR-29b/GO-PEG-PEI@chitosan hydrogel that shows excellent miR-29b loading capacity and delivery efficiency. In vivo experiments showed that transfection of BMSCs significantly promoted their osteogenic differentiation and did not trigger inflammatory responses. Kim et al. [175] coupled protamine with poly(organophos-phazene) and loaded siRNA nanocomplexes onto it to create a nanohydrogel that was injected into the organism. The loaded siRNA complexes crossed the cellular barrier to achieve high efficiency of delivery and reduce the expression of the corresponding genes and proteins. Furthermore, NPs containing RNA therapeutic agents (such as miRNAs, siRNA, and siRNAs) are embedded in the hydrogel matrix, which is then implanted at the tumor site for tumor gene therapy [176]. However, there are challenges and problems associated with this combination strategy. First, the combination of nanomaterials and hydrogels may affect their biocompatibility and drug-release properties. Second, this combination may increase the complexity and cost of treatment. More research is needed to validate the safety of this binding strategy for the treatment of tissue trauma.

### Microspheres loaded with genes and combined with hydrogels

Microspheres are a class of tiny spheres formed by the dissolution of drugs in polymeric materials, with a particle size of 1–250 μm, suitable for gene encapsulation or adsorption on their surface to ensure slow drug-release efficiency [178]. They have low toxicity, are degradable and safe, have a special affinity for some cell tissues, and can be endocytosed by the reticuloendothelial system (RES) to achieve targeting. Moreover, the route of administration of microspheres is mainly injection. This can bypass the first-pass effect, improve utilization, and reduce the dose of administration (Fig. 6B) [177]. Phase separation, hot-melt extrusion, emulsification, volatilization, and spray drying are common methods of microsphere preparation [179, 180]. Microspheres play an important role in numerous fields. Gan et al. [181] used MSC-derived exosomes (MSC-Exos) externally encapsulated in SA hydrogel microspheres to protect against degradation in the intestinal environment as an agent to treat intestinal inflammation. Furthermore, the microspheres reduced the levels of pro-inflammatory cytokines in damaged colonic epithelial cells and inflammatory macrophages, which is highly effective in treating acute colitis. DiStefano et al. [182] embedded MSC-Exos in a poly(lactic-co-glycolic acid) microsphere and combined it with a hydrogel, a system that sustainably delivers MSC-Exos for fibrotic repair under the protection of cells from a denaturing and pro-inflammatory environment. The system stimulates cell proliferation and migration by promoting sustained treatment of IVDD by promoting stimulation of cell proliferation and migration. The combination of microsphere-loaded genes with hydrogels represents an advanced strategy for creating hydrogel gene therapy systems with adaptive and self-healing properties. This system has the advantage of enabling sustained and controlled gene release while safeguarding them from the in vivo environment. Hydrogels can also be used as reservoirs for drugs and genes to further enhance their therapeutic effects.

The release of therapeutic genes cannot be separated from the carriers, and smart-responsive hydrogels are the preferred design strategy. Some hydrogels are characterized by high sensitivity and an adjustable structure, especially physically cross-linked hydrogels that can respond to external stimuli by changing the pathways of swelling, contraction, and sol–gel phase transition to achieve targeted gene release at specific sites. However, there is a need to emphasize the improvement in controlling environmental stimuli in some performances [72]. Microspheres/nanomaterials loaded with genes and then combined with hydrogels are a key design strategy that increases the gene-carrying capacity, protects the genes, and facilitates the slow release of genes. However, the microsphere production process is complicated, the sterilization cost is high, the encapsulation rate is low, and the safety of the nanomaterials and the preparation cost must be considered. Therefore, the release of therapeutic genes needs to be further investigated. We hope that with continued scientific research these difficulties can be solved.

## Tissue engineering applications

Hydrogel biocompatibility, encapsulability, and plasticity are suitable for repair after tissue damage. This review focused on gene therapy hydrogels within bone regeneration, cardiac repair, cartilage repair, spinal cord injuries, skin trauma, eye disorders, tumor treatment, osteoarthritis, brain disorders, inflammatory bowel disorders, Rheumatoid arthritis, and hair regeneration. The delivery gene, hydrogels, and their therapeutic effects in tissue engineering applications are shown in Table 4.

**Table 4:** Delivery gene, hydrogels, and their therapeutic effects for tissue engineering applications

Application	Delivery gene	Hydrogel	Effect	Ref
Bone regeneration	miRNA	MAHSs	Accelerates osteogenic differentiation	[52]
		miR-29b/GO-PEG-PEI@Chitosan	Promotes osteogenic differentiation	[174]
		Exos-HA	Achieves sustained release of miRNA antagonists to accelerate osteogenesis	[117]
		uMSCEXOs/Gel/nHP	Accelerates the repair of cranial bone defects	[183]
Heart repair	Exos	MA-HA	Induces the proliferation and differentiation of epicardial-derived cells	[95]
		Gel@Exo	Reduces the fibrotic area	[184]
	miRNA	HA	Inhibition of Hippo signaling	[185]
Cartilage repair	miRNA	MS@G5-AHP/miR-140	Prevents degeneration of articular cartilage and relieves symptoms of OA	[161]
		Nanofiber	Slows down the aging of chondrocytes	[186]
	Exos	H-Exos	Repairs injured cartilage	[187]
Spinal cord repair	miRNA	H-Exos	Activates the PI3K/AKT signaling pathway	[188]
	Exos	USC-Exo	Repairs spinal cord injury	[189]
Skin trauma	Exos	SS-SF	Accelerates blood vessel regeneration	[56]
		PEG	Induces reprogramming of M1-Mφs to M2-Mφs	[64]
	siRNA	siRNA-TGFβ1-337	Promotes apoptosis of scar fibroblasts	[170]
		Functional peptide hydrogels	Anti-ad effect and intradermal penetration	[190]
Eye disease	siRNA	siVEGF NB	Bypasses the TLR3-induced siRNA-like effector pathway to treat AMD	[177]
Tumor treatment	siRNA	Polyethylenimine	Controlled slow release of polyplex	[152]
Osteoarthritis	Exos	CS	Accelerates ECM remodeling and defective regeneration of cartilage	[47]
	miRNA	MS@G5-AHP/miR-140	Alleviates degradation of the cartilage matrix, inhibits expression of MMP5 and MMP13	[161]
Brain disease	Exos	FPGEGa	Promotes nerve regeneration in TBI rats	[191]
Enteric diseases	siRNA	SA@MOF-siRNATNFα	Reduces the condition of enteritis	[192]
Rheumatoid arthritis	siRNA	Anti-RelA siRNA	Reduces the incidence and symptoms of RA	[29]
	Exos	Exos@SFMA	Prevents joint damage and relieves synovial inflammation	[193]
Hair regeneration	miRNA	SMSC-126-Exos	Activates Wnt/β-catenin channels to promote hair regeneration	[194]
		HA	Promotes hair regrowth	[156]

*nHP* nanohydroxyapatite/poly-ε-caprolactone, *OA* osteoarthritis, *AMD* age-related macular degeneration, *TBI* traumatic brain injury, *RA* rheumatoid arthritis

### Bone regeneration

**Fig. 7:**
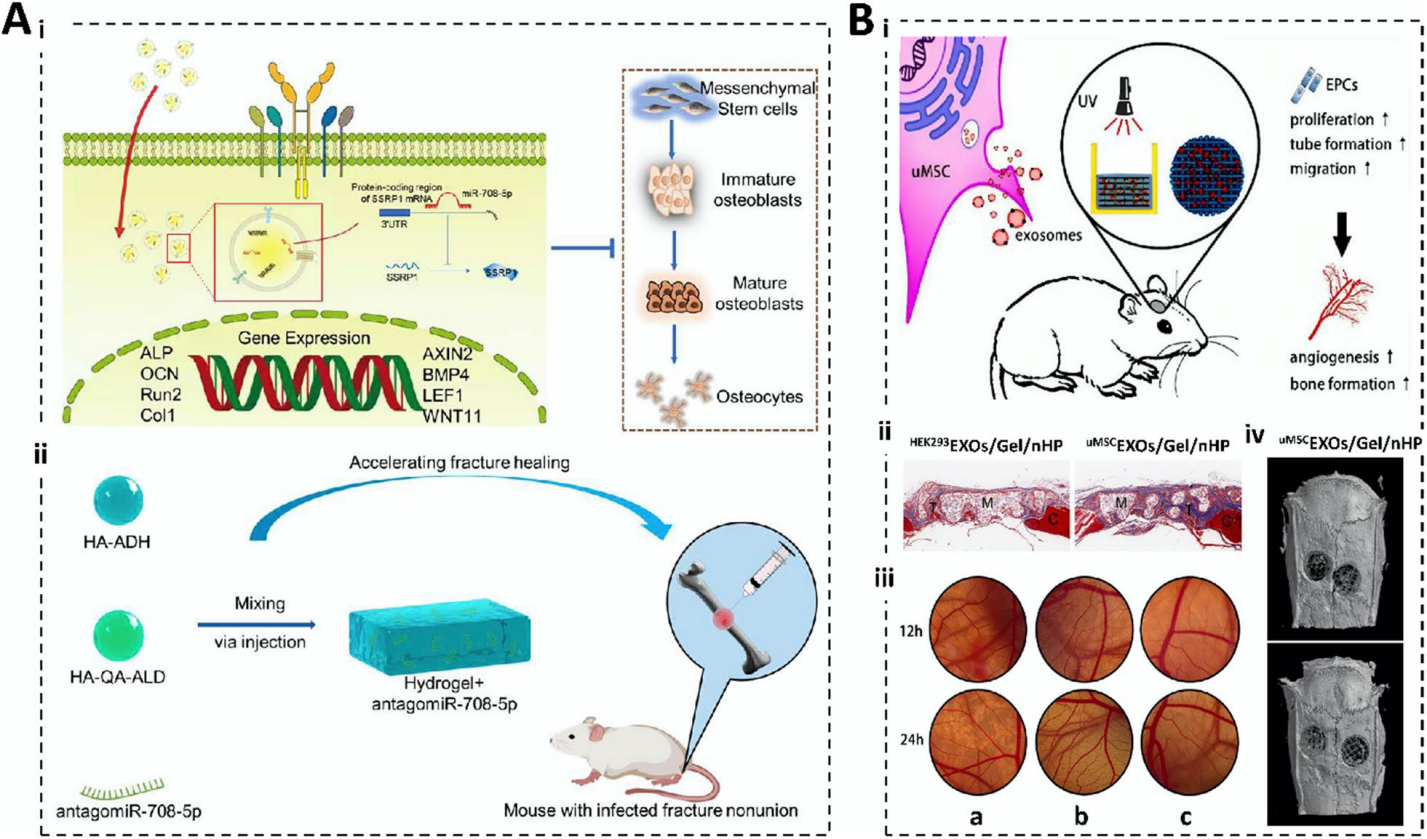
Applications of therapeutic gene hydrogels in bone regeneration. **A** Effect of Exos hydrogel on osteogenesis of bone marrow mesenchymal stem cells. (i) Exosomal miR-708-5p inhibits osteogenic differentiation of BMSCs; (ii) Exos hydrogel to treat infected bone nonunion. (Reproduced with permission from Ref. [117], Copyright 2022, American Chemical Society). **B** Accelerated bone repair in rats with uMSC-derived Exos. (i) Schematic diagram of uMSC-derived Exos accelerating bone repair in rats; (ii) Masson’s trichrome staining; (iii) promoting neovascularization; (iv) realizing bone repair. (Reproduced with permission from Ref. [183], Copyright 2021, American Chemical Society)

An increasing number of patients are experiencing fractures, bone defects, and osteoporosis, often owing to trauma, car accidents, and tumor ablation [195]. A fracture is defined as a fracture of the bone caused by trauma or pathology, presenting with clinical manifestations such as localized pain, swelling, and ecchymosis. Stem cell and biofactor therapies are commonly used for these conditions [196]. Demineralized bone matrix (DBM) is a biomaterial produced by removing the mineral content of the bone and is used to repair fractures and bone defects. However, commercially available DBM products often employ calcium sulfate, SA, and HA as delivery vehicles. These substances can spread throughout the body, causing inflammation and diminishing bone regeneration [197]. Studies have highlighted the effectiveness of Exos hydrogel in treating fracture nonunion. The hydrogel’s excellent adjustability and hydrophilicity make it suitable for mimicking the natural bone tissue environment. Yu et al. [117] demonstrated sustained release of miRNA antagonists using a sensitized hydrogel, incorporating Exos loaded with antagomiR-708-5p onto HA hydrogels to treat femoral fracture nonunion in mice. AntagomiR-708-5p promotes the Wnt/β-catenin signaling pathway, enhancing the osteogenic differentiation of organelle cells. Compared with the blank group, the hydrogel exhibited improved antibacterial and anti-inflammatory properties, biocompatibility, and mechanical support, leading to enhanced bone differentiation and accelerated fracture healing (Fig. 7A).

Bone regeneration is influenced by factors such as osteoblasts, osteogenic spiking factors, and osteogenic scaffolds. The HA hydrogel is an outstanding osteogenic scaffold [42, 198]. Exosomes contain a variety of RNAs and are a friendly osteogenic factor [16]. Zhang et al. [183] developed uMSCEXOs/Gel/nanohydroxyapatite/poly-ε-caprolactone (nHP) hydrogels by wrapping uMSCEXOs on an HA-Gel modified with nHP. In a rat cranial bone defect model, the gel induced increased expression of angiogenesis-promoting factors, such as VEGFA and HF-1α, through upregulation of the NOTCH1/DLL4 pathway, which promotes vascular neovascularization and osteogenic differentiation. The hydrogel also promoted endothelial progenitor cell proliferation and angiogenesis, providing strong mechanical support for improved bone regeneration (Fig. 7B). However, gene therapy hydrogels must meet a number of stringent requirements if they are to be effective in the field of bone regeneration. These include suitable biocompatibility, sufficient mechanical strength, excellent osteogenic properties, excellent integration ability, and long-lasting slow drug release. In particular, hydrogels must meet certain standards in terms of mechanical strength, otherwise, it will be difficult to meet the needs of the bone regeneration field. Therefore, the application of gene therapy hydrogels in the field of bone regeneration still needs to undergo in-depth research and exploration to promote the progress of material science and bring substantial benefits to patients.

### Heart repair

Heart injury is abnormal damage to the structure or function of the heart, blood vessels, nerves, and other tissues caused by external or internal factors [199]. Common manifestations include MI, heart valve disease, cardiomyopathy, coronary artery disease, post-surgical cardiac adhesions, and other related diseases [200]. Although medication, surgery, and intervention are commonly used repair methods, some drugs may cause side effects with persistent effects on the organism. Gene therapy hydrogels offer a solution by achieving stable and continuous delivery of therapeutic genes to the target location to repair injuries [201]. Zhu et al. [184] prepared MA-HA hydrogels with methacrylic anhydride (MA)-cross-linked HA and loaded Exos secreted by MCSs onto the hydrogels. Exos injected through iPC had a prolonged residence time in the cardiac organ, inducing the proliferation and differentiation of epicardial-derived cells, increasing epicardial thickness, and reducing fibrosis after MI.

Myocardial infarction (MI) results from myocardial hypoxia and ischemia owing to coronary blood flow obstruction leading to necrosis of myocardial tissue. Its treatment focuses on promoting functional recovery of the myocardium and local blood circulation [202]. Wang et al. [185] developed a miR-302-targeted HA hydrogel for cardiomyocyte proliferation and repair after cardiac ischemic injury. The hydrogel combined large tumor suppressor 2 and macrophage-stimulating 1 to inhibit Hippo signaling and activate the expression of cell proliferation-related genes. Spectral tracing, fluorescence scanning images, and clonal cell analysis showed that the MI mouse hearts injected with the hydrogel reactivated the cell cycle of cardiomyocytes and promoted its sustained proliferation, which is suitable for cardiac regeneration and improving related problems after MI. In conclusion, gene therapy hydrogels emerge as an excellent option for heart repair owing to their minimally invasive, long-lasting, and relatively safe nature. Non-toxicity is a prerequisite for cardiac repair, and good biocompatibility is essential to prevent immune rejection. The therapeutic gene hydrogel should offer suitable mechanical support, with characteristics similar to those of rheological suitability, ensuring that it does not excessively affect blood flow. As medical advances continue, there is hope for a new era in heart repair.

### Cartilage repair

Cartilage is a translucent, elastic, and tough tissue that plays a supportive and protective role in organisms [26]. There are several causes of cartilage damage, including ischemia, traumatic injury, and degenerative diseases [203]. Osteoarthritis (OA) causes cartilage cells in the joints to age, causing cartilage damage [42, 204]. miR-29b-5p upregulates TET1 to repress the expression of P16INK4a/P21, senescence-associated genes, and MMPs, slowing chondrocyte senescence and repairing defective cartilage by improving the microbial milieu [205]. Zhu et al. [186] loaded agomir-29b-5p onto a self-assembled nanofiber hydrogel to induce the homing of MSCs. The anterior cruciate ligament transection in rats showed that the hydrogel had outstanding mechanical properties, good biocompatibility, moderate pore size, and could slow chondrocyte senescence through the P16INK4a/P21 pathway. Zhang et al. [47] prepared an injectable alginate-dopamine (AD), CS, and regenerated silk fibroin (AD/CS/RSF) hydrogel that has strong adhesion and can efficiently promote efficient proliferation of BMSCs after loading with Exos. This is an innovative strategy for the minimally invasive treatment of cartilage defects.

Exosomes contain various nucleic acids commonly used for cell proliferation, cartilage repair, and other purposes. Bone marrow stem cell-derived Exos can secrete trophic factors such as TIMPs and TFG-β through a paracrine mechanism to promote articular chondrocyte functionalization [206]. Although TEM did not reveal morphological differences between hypoxia-preconditioned exosomes (H-Exos) and normoxia-preconditioned exosomes (N-Exos), the former was more easily internalized in articular chondrocytes and more effective in promoting migration, proliferation, anti-inflammation, and matrix synthesis. Furthermore, it upregulated Exos miR-205-5p expression, efficiently activated the PTEN/AKT pathway, inhibited the expression of RUNX2, inhibited inflammation, promoted chondrocyte proliferation, migration, and metabolism, and repaired defective cartilage [207]. However, Exos are locally administered and easily removed. Shen et al. [187] modified the H-Exos hydrogel to achieve an efficient and sustainable release of Exos and achieved significant results in the repair of cartilage defects in rat models. Hydrogels as carriers for gene therapy can protect and deliver genes, ensuring that they act on damaged cartilage.

### Spinal cord injury

The spinal cord located within the spinal column is an important neural structure that connects the brain to the trunk, transmits information, and coordinates body functions. Spinal cord injury (SCI) causes neurons and nerve fibers to break down, resulting in the inability of nerve signals to be properly transmitted, affecting the body’s sensory, motor, and autonomic functions of the body [208]. It can be subdivided into primary and secondary SCI, with the first being damage owing to external forces acting on the spinal cord and the second being further damage to the spinal cord resulting from compression of the spinal cord owing to hemorrhage of small blood vessels in the spinal canal to form hematomas, spinal cord edema, compression fracture, and fragmentation of intervertebral disk tissue [209]. Bone MSC-derived exosomes were used as therapeutic agents for SCI. Cheng et al. [210] prepared a GelMA-Exo delivery system by loading Exos with GelMA, which significantly improved Exo retention, promoted neuron differentiation and extension, induced neurogenesis, and significantly reduced scar formation.

Bone marrow stem cell-derived H-Exos-encapsulated miR-216a-5p repairs traumatic spinal cord by inducing glial cell M1/M2 polarization to sustain inhibition of TLR4/NF-κB and activation of PI3K/AKT signaling pathway [188]. Pluronic F-127 hydrogel-mediated shRNA induces the proliferation of neuronal and myeloid cells by inhibiting Lingo-1 expression after bone marrow fracture in rats [211]. The USC-Exo hydrogel injected into the SCI model crossed the spinal cord blood–brain barrier and delivered ANGPTL3 to damaged areas. Therefore, it mediates the PI3K/AKT signaling pathway, promotes vascular regeneration, and restores the neurological function of the spinal cord [189]. miR-124 is associated with pro-neuronal differentiation of neural precursor cells, which inhibit inflammatory cytokine production and prevent deterioration of the damaged spinal cord. Louw et al. [212] intraperitoneally injected chitosan hydrogel with miR-124 into rat SCI, which significantly inhibited the expression of MHC-II and TNF-α. Reduced ED-1 macrophage content in damaged spinal cord inhibits secondary neuronal damage and inflammatory effects caused by microglia/macrophage secretion proteins.

Gene therapy hydrogels are an effective SCI treatment strategy, whereby the delivery of the target gene to the targeted location is achieved with the advantageous action of the hydrogel, leading to treatment of the spinal cord treatment. The selection of appropriate genes (such as exosomes or miRNAs) that promote nerve cell growth and connectivity for better functional recovery of the damaged spinal cord is critical for spinal cord therapy. Safety, long-term efficacy, monitoring, and biosolubility are also issues of concern.

### Skin trauma

**Fig. 8:**
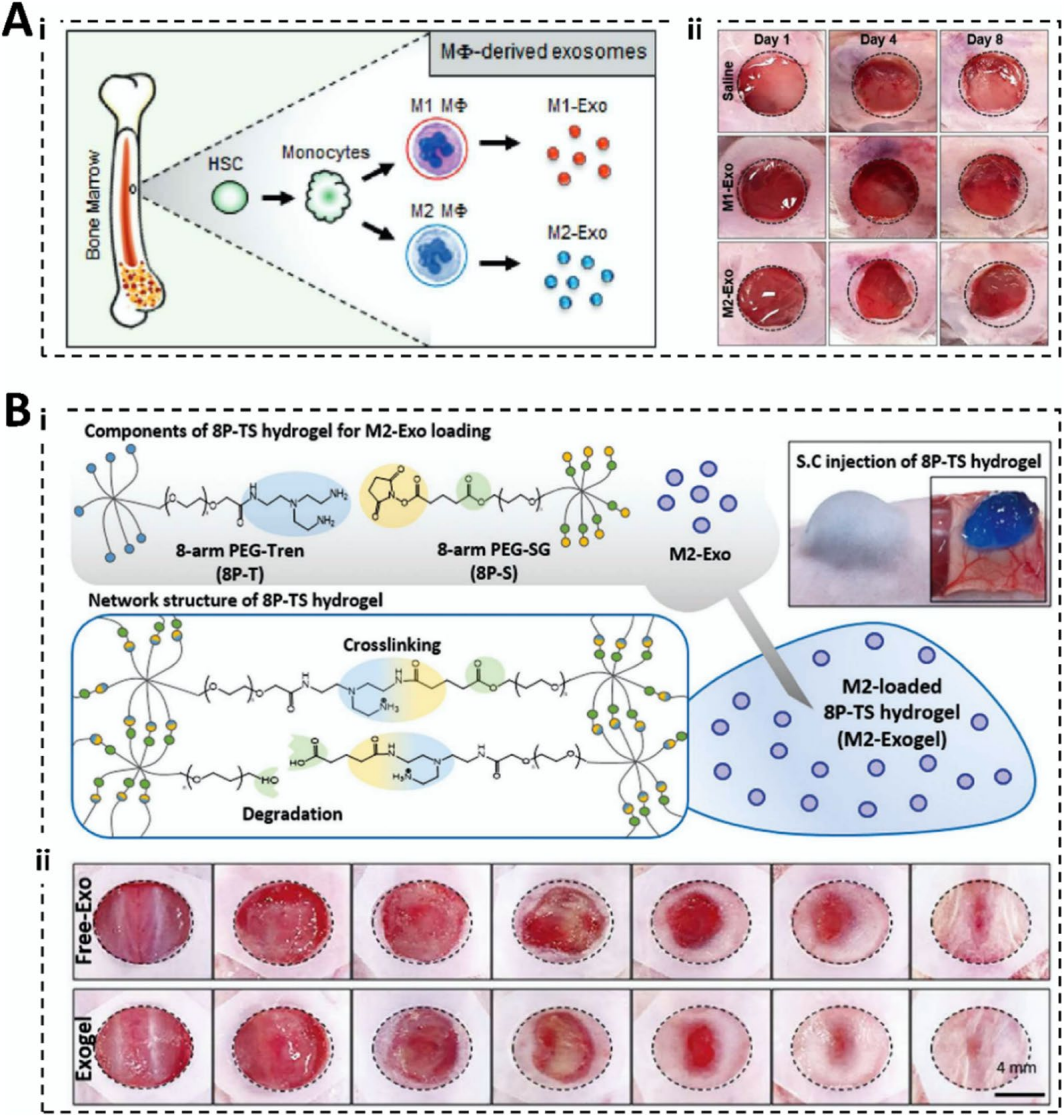
Applications of therapeutic gene hydrogels in skin trauma. **A** Exosomes promote skin wound healing. (i) Schematic diagram of Exos and macrophage (ϕs) preparation; (ii) promotion of wound healing. (Reproduced with permission from Ref. [214], Copyright 2019, John Wiley and Sons). **B** The 8P-TS Exogel was used for wound repair. (i) Schematic diagram of the gel formation strategy for hydrogels; (ii) accelerated wound healing. (Reproduced with permission from Ref. [64], Copyright 2022, John Wiley and Sons)

The skin comprises three layers: the epidermis, dermis, and subcutis. It is the largest organ of the body, covering the surface of our body and protecting it from the environment [36, 213]. However, the human body often experiences skin trauma owing to external forces. Medical advances have led to the development of effective treatments for skin trauma. Gene therapy hydrogel is an emerging treatment method with the advantage of realizing the efficient delivery of therapeutic genes and inducing the regeneration of damaged skin (Fig. 8A) [214]. Exosomes derived from human adipose-derived MSCs (hADSCs) are effective for skin trauma and improve Exo retention after in vivo transplantation. Zhou et al. [215] used the heat-sensitive Pluronic F-127 hydrogel to encapsulate hADSCs-Exos to promote wound healing and regeneration, which significantly increased the expression of α-SMA, CD31, and Ki67, and reduced the inflammatory response by upregulating the expression of AQP3 and KRT1 skin barrier proteins. This led to the repair of the skin wounds.

Efficient and sustained delivery of siRNA is necessary for skin trauma repair. Therefore, a hydrogel containing anti-RelA siRNA and functional peptides was prepared. This hydrogel showed anti-atopic dermatitis effects and intradermal penetration in an AD mouse model and improvement in severe skin trauma [190]. Cao et al. [216] prepared a composite hydrogel that mimicked the ECM, provided attachment points and nutrients to cells, and promoted VEGF expression. The hydrogel exhibited good skin regeneration in terms of total skin defects. During trauma healing, macrophages (Mφs) shift from pro-inflammatory (M1) to anti-inflammatory (M2), which is critical for acute inflammation relief and guided tissue repair. Kwak et al. [64] extracted Exos (M2-Exos) from M2-Mφs and used them as local microenvironment signals to induce reprogramming of M1-Mφs to M2-Mφs. Sustained results were achieved by designing a PEG hydrogel and using it to piggyback on M2-Exos to maximize the rate of skin wound healing. Localized M2-Exos can induce a rapid transition of M1 macrophages to the M2 state in damaged and diseased skin tissues, thus accelerating the rate of damage. These benefits include faster wound closure and better healing rates (Fig. 8B).

Hydrogels perform exogenous debridement and promote autolytic debridement during skin wound repair. Simultaneously, hydrogels can improve the regeneration of wound granulation tissue, promote epithelial cell division and migration, and accelerate wound healing. In terms of wound healing transformation, gene therapy is able to promote wound healing from the source by directly regulating cell growth, differentiation, and metabolic processes. In addition, a patient’s wound healing process is unique and can be interfered with by a variety of factors such as environment, genetics, and age. Gene therapy allows for a personalized treatment plan, thus providing a more precise and effective treatment. Traditional wound treatment often faces problems such as rapid degradation and uneven release of drugs [43]. A hydrogel, as a drug delivery system, can effectively protect the gene drug from the damage of the external environment, and at the same time achieve a continuous and stable release of the drug. This can not only improve the utilization rate of the drug but also prolong its action time, thus enhancing the therapeutic effect. The biocompatibility and degradation rate of hydrogels are key factors that limit their application [155]. Although hydrogels are designed to mimic the microenvironment of the extracellular matrix, their biocompatibility is not always perfect. In some cases, hydrogels may cause an immune or inflammatory response that can interfere with the normal wound-healing process. If the degradation rate of the hydrogel is too fast, it may not provide adequate support to the wound; if the degradation rate is too slow, it may hinder the formation of new tissue and wound healing. The stability of therapeutic genes in hydrogels is affected by a variety of factors, such as temperature, humidity, and pH. If the treatment is degraded or inactivated in the hydrogel, the therapeutic effect will be greatly reduced. Therefore, how to ensure the stability and activity of therapeutic genes in hydrogels is an urgent problem to be solved.

### Eye diseases

**Fig. 9:**
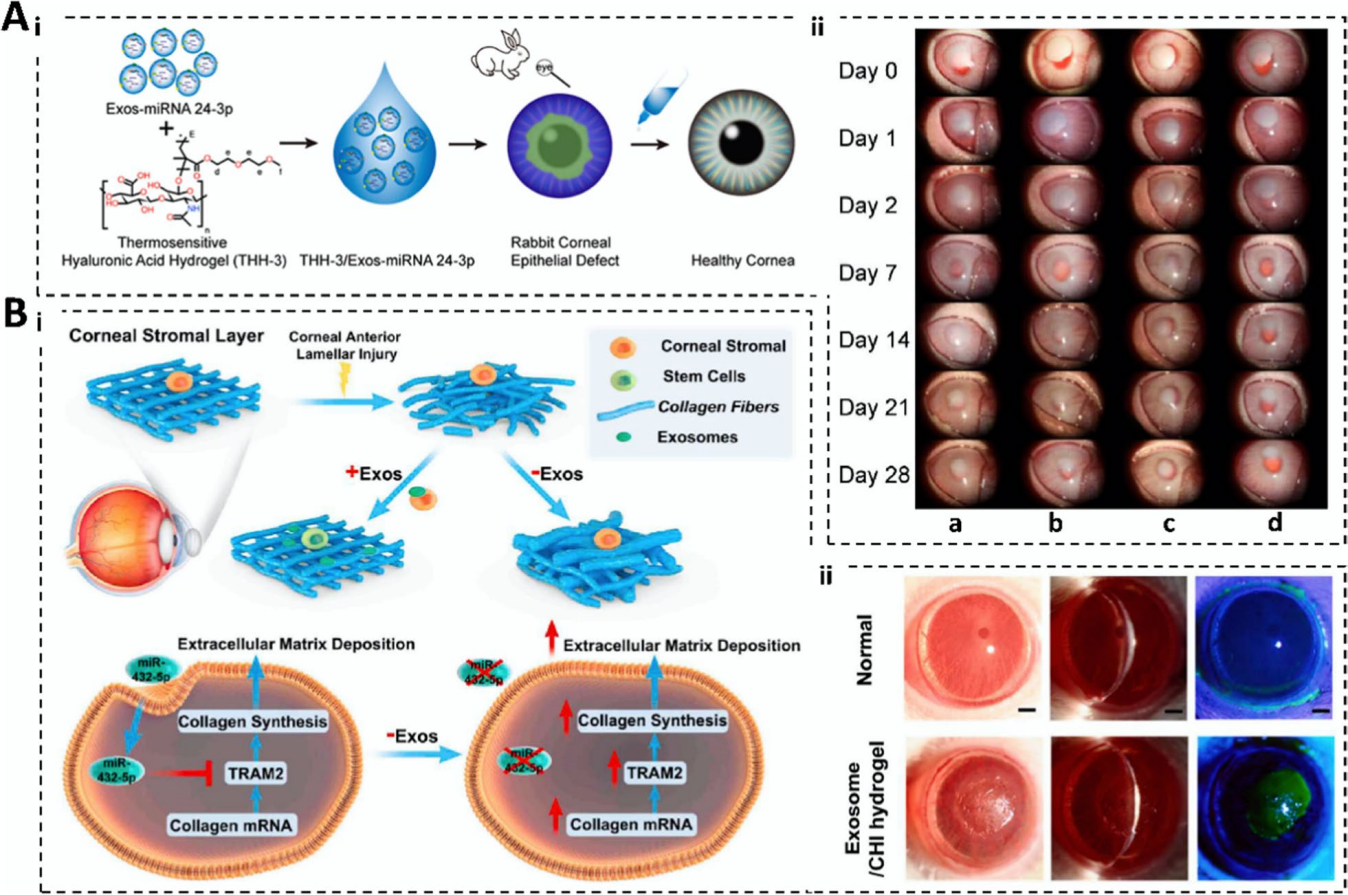
Applications of therapeutic gene hydrogels in eye diseases. **A** HA hydrogel loaded with miRNA 24-3p exosome promotes corneal epithelial healing. (i) Exos-miRNA 24-3p hydrogel for repair after corneal epithelial defects; (ii) Evaluation of in vitro release of Exos-miRNA in a rabbit corneal epithelial defect model. (Reproduced with permission from Ref. [16], Copyright 2022, Elsevier). **B** Temperature-sensitive hydrogel regenerates corneal epithelium after piggybacking on Exos. (i) Extracellular matrix (ECM) remodeling after anterior corneal plate injury; (ii) Repair of the anterior corneal lamina propria by the CHI hydrogel. (Reproduced with permission from Ref. [218], Copyright 2022, Elsevier)

The eye is an important part of the visual system of humans and animals, and is the main organ that senses the outside world. The eye consists of the eyeball, cornea, pupil, lens, and retina, among others. Therefore, the organizational integrity of the eye’s component structures is a prerequisite for visual function. However, eye diseases often occur due to excessive and inappropriate eye use and trauma [217]. With the growing interest in gene therapy, hydrogel materials, and tissue engineering techniques, and also based on the advantages of high water retention and stability of hydrogels, gene therapy hydrogels are a very advanced strategy for the treatment of eye diseases. Exosomes promote corneal epithelial healing. Sun et al. [16] prepared a THH-3/Exos-miRNA 24-3p hydrogel that improves corneal epithelial cell migration and induces post-healing of corneal epithelial defects, effectively preventing macrophage activation and corneal stromal fibrosis (Fig. 9A). Tang et al. [218] used temperature-sensitive chitosan hydrogels to sustain the release of iPSC-MSC-derived Exos to induce repair of the stromal layer and corneal epithelial cells and downregulate the mRNA expression of collagen to reduce scar formation. Furthermore, the hydrogel inhibited TRAM2 to inhibit ECM deposition, which is effective for corneal repair after injury (Fig. 9B). Effective control of the gene therapy process can be achieved through precise control of the temperature to improve therapeutic effects since both hydrogels are temperature-responsive.

Age-related macular degeneration (AMD) is an eye disease that affects the central vision and is associated with choroidal neovascularization (CNV). Cells and tissues in the macular region of patients with AMD gradually degenerate, leading to blurred vision and blindness [177]. siRNA therapy is indicated for this class of diseases, but its efficacy is limited by a short working time, TLR3-related undesirable pathway activation, and poor delivery to the desired subretinal target tissue [219]. Ryoo et al. [212] prepared an anti-VEGF nanoball (siVEGF NB) encapsulated by an siRNA hydrogel. In a CNV mouse tissue model, the nanospheres overcame the vitreous to enter the retina and subchoroidal space through endocytosis of CD44 receptors on the inner rim, bypassing the TLR3-induced siRNA-like effector pathway, thereby showing good tissue therapeutic effects. The DNA-based composite hydrogel system supports the water-soluble ophthalmic treatment of allergic conjunctivitis [220].

In conclusion, gene therapy hydrogels have powerful therapeutic effects on eye diseases and can provide new methods and ideas to treat various eye diseases. Gene therapy safety and biocompatibility are crucial. Therefore, clinicians should pay special attention to the characterization of their composition, crosslinking degree, and degradability when selecting and using hydrogels. Temperature-sensitive hydrogels may gain popularity in the treatment of eye diseases.

### Tumor treatment

A tumor is a disease caused by the abnormal proliferation of cells in the body owing to gene mutations, which can be classified as benign or malignant [221]. Benign tumors grow slowly, are not easy to metastasize, are surrounded by a peripheral membrane made of proliferating connective tissues, have obvious boundaries with the surrounding tissues, do not cause pressure and damage to the surrounding tissues, and generally have little effects; only in vital organs (intracranial, thoracic cavity) will they threaten life. Malignant tumors grow rapidly, have no envelope, the boundary between tissues is blurred, and the tumor cells will spread to the surrounding tissues and invade the interstices, ducts, and cavities of the surrounding tissues, which will cause compression and damage to the neighboring organs and tissues [222]. Local infiltration and distant metastasis are the most important characteristics of malignant tumors and are the main causes of death from malignant tumors [223]. Tumors can be treated using a variety of methods, such as surgery, radiation therapy, and chemotherapy [223]. Paclitaxel is an excellent anticancer agent that effectively induces apoptosis and inhibits the Akt1 signaling pathway and the growth of human papillary carcinoma cells [224]. The MMP-sensitive PEG hydrogel vectors had a significant sensitizing effect on tumor necrosis factor α-related apoptosis-inducing ligand (TRAIL) when mediating glioblastoma (GBM) cells in synergizing with the induction of apoptosis by GBM [225]. Hydrogels can carry therapeutic genes for local delivery to attack primary tumors or inhibit primary tumor recurrence, which is a novel tumor therapy strategy.

siRNA hydrogels exert antitumor effects by silencing the cell cycle protein B1 [152]. Peng et al. [226] used collagen hydrogels as carriers to achieve local, efficient, and sustained targeted delivery of Id1-targeted siRNAs, confirming the feasibility of their use for gastric cancer inhibition. Nanoparticles containing RNA therapeutic agents (such as miRNAs, siRNAs, and siRNAs) were embedded in hydrogel matrices, which were then implanted near the tumor for tumor gene therapy [176]. However, excessive hydrogel crosslinking can hinder the release of RNA therapeutic agents and reduce their therapeutic efficiency. Therefore, single-component injectable RNA hydrogels are preferred [227]. RNA hydrogels can manipulate immunomodulatory factors and silence oncogenes during cancer immunotherapy [102, 173].

In conclusion, gene therapy hydrogels are excellent materials for tumor treatment. However, numerous factors must be considered, such as tumor size, location, and morphology, as well as the effect of injection on surrounding tissues and organs. Furthermore, the injection route is limited by the type and distribution of the tumor. Moreover, clinicians should ensure that hydrogels are compatible and coordinated with other therapeutic approaches (such as surgery, radiotherapy, or chemotherapy) to improve treatment efficacy before using gene therapy hydrogels as part of a comprehensive treatment strategy.

### Osteoarthritis

**Fig. 10:**
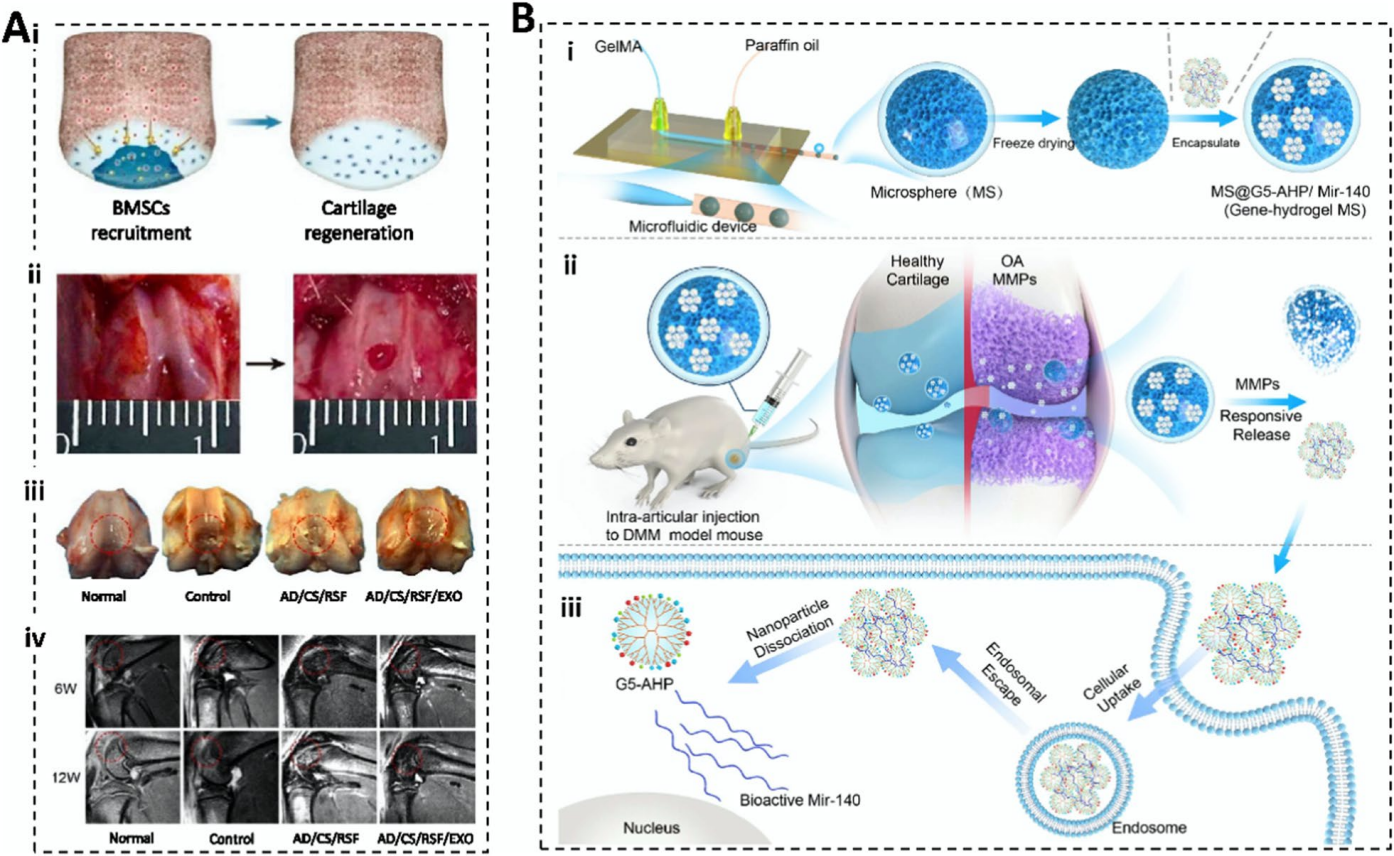
Applications of therapeutic gene hydrogels in OA. **A** Injectable exosome hydrogel for cartilage defect regeneration. (i) Promoting superficial cartilage regeneration; (ii) Repair of cartilage defects by the AD/CS/RSF/EXO hydrogel; (iii) Gross observation of cartilage integrity; (iv) MRI images of rat knees. (Reproduced with permission from Ref. [47], Copyright 2021, Elsevier). **B** MS@G5-AHP/miR-140 to treat OA. (i) Gene-hydrogel MSs; (ii) Articular cavity injection of MS@G5-AHP/miR-140 to alleviate OA; (iii) Release of miR-140 and endocytosis of G5-AHP/miR-140. (Reproduced with permission from Ref. [161], Copyright 2022, Springer Nature)

Osteoarthritis (OA) is a non-inflammatory and degenerative joint disease that is more common in people over 50 and women, with symptoms of joint stiffness, pain, and swelling [228]. The main pathologies include cartilage degeneration or disappearance, joint limb ligament attachment, and cartilage-bone hyperplasia to form osteophytes [229]. Zhang et al. [47] prepared a composite hydrogel with enbucrilate modified with regenerated SF, CS, and AD, and its cross-linked network was highly bound to the wet surface, demonstrating high adhesive strength and biocompatibility. The loaded exosomes efficiently promoted the proliferation, differentiation, expansion, and migration of BMSCs. In situ injection of the hydrogel into the rat patellofemoral groove released exosomes that recruited BMSCs into newborn cartilage through the chemokine signaling pathway, accelerating the rate of the remodeling of ECM and OA treatment (Fig. 10A). The cause of OA is unknown but may be related to obesity and aging. Excess joint activity, joint trauma, intraosseous hypertension, and osteoporosis can lead to the development of OA. In the early stages of OA, the surface layer of the body is deficient owing to cartilage degradation and loss of adhesion to wet tissue, resulting in a lack of cartilage regeneration [230].

Osteoarthritis is a non-inflammatory degenerative disease caused by tissue degeneration, chronic injury, and reduced cartilage elasticity, and is caused by increased production of MMPs [231]. Hydrogels are injectable, highly compatible, and of great interest for minimally invasive tissue repair. Amino acid coupling improves gene transfection efficiency [160]. Li et al. [161] constructed an MS@G5-AHP/miR-140 hydrogel that exhibits a high transfection efficiency for the release of G5-AHP/miR-140 nanoparticles. It maintains the metabolic balance of the cartilage matrix, alleviates cartilage matrix degradation, and inhibits the overexpression of MMP5 and MMP13. The hydrogel prevents articular cartilage degeneration, reduces osteophyte formation, and alleviates OA symptoms. It also compensates for the short half-life, low transfection rate, and susceptibility to inactivation caused by direct injection of crude miRNAs; this demonstrates the excellent therapeutic ability of gene therapy hydrogels (Fig. 10B).

The advantage of gene therapy hydrogels is that they can be injected into the joint through a minimally invasive procedure with sustained and controlled release of the agent at the implantation site. This local delivery is ideal for patients with OA since it reduces systemic side effects and can be personalized according to the specific needs of each patient.

### Brain diseases

**Fig. 11:**
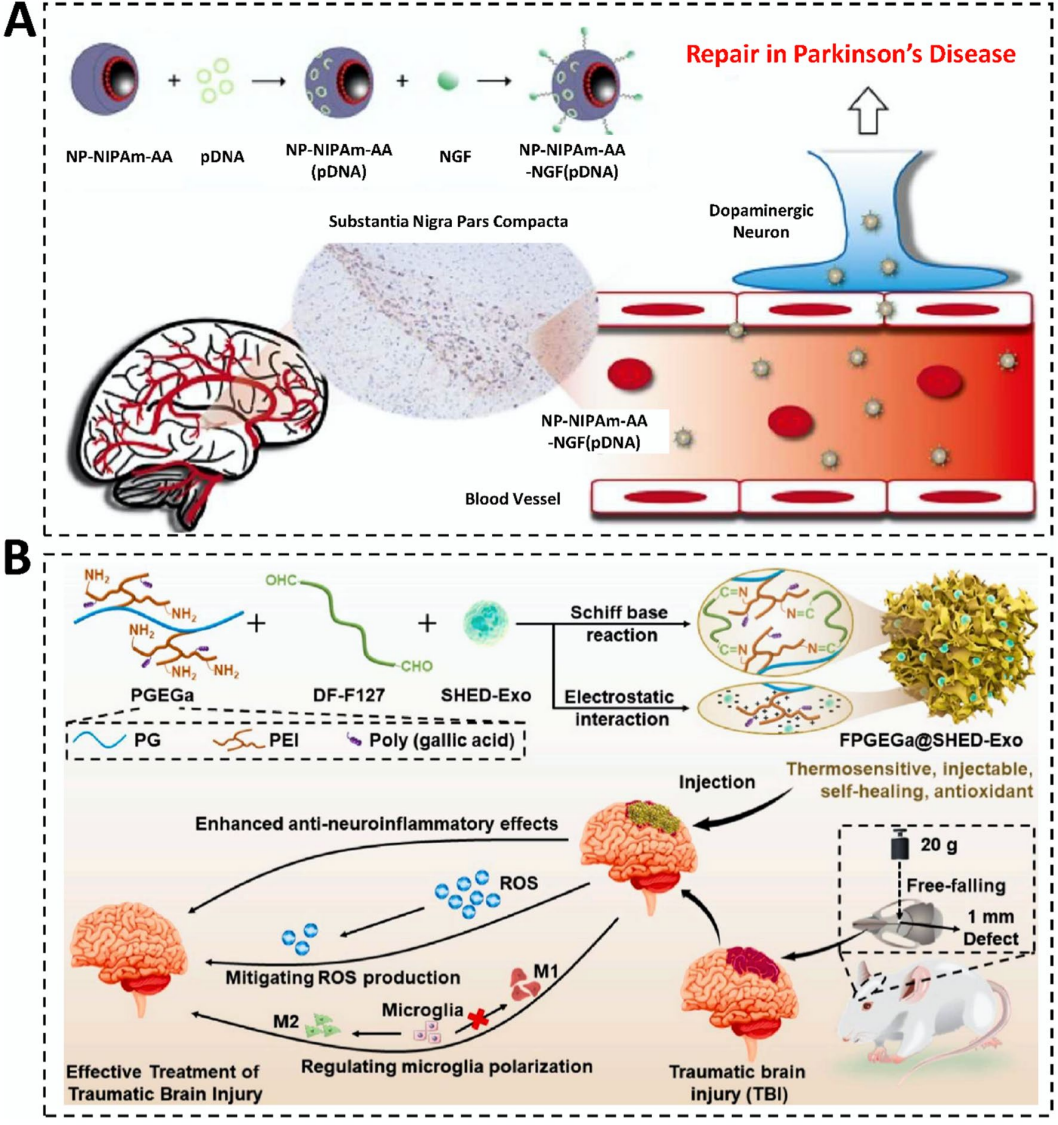
Applications of therapeutic gene hydrogels in brain diseases. **A** Schematic representation of the inhibitory effect of RNAi plasmid on Parkinson’s disease. (Reproduced with permission from Ref. [236], Copyright 2017, Ivyspring). **B** Exosomal hydrogels and microglia work together for nerve repair after TBI. (Reproduced with permission from Ref. [191], Copyright 2022, Elsevier)

Brain diseases are commonly caused by inflammation of intracranial tissues and organs (cerebrum, cerebellum, brainstem, cranial nerves, and meningeal blood vessels), vascular diseases, parasitic diseases, and trauma [232]. The extrapyramidal system is an integral part of the locomotor system, and the striatal pallidum is the main structure of the extrapyramidal system. Structures of the thalamic floor nucleus, red nucleus, substantia nigra, and reticularis are also included. Damage to the striatal pallidocellular system causes hypertonic hypokinesia and hypotonic hyperkinesia syndrome. The former is like Parkinson’s disease (PD) and involves lesions located in the substantia nigra and nigrostriatal pathways, whereas the latter is like chorea, bradykinesia, and torsion spasticity, and involves lesions located in the striatum [233, 234]. Hydrogels combined with MSCs, and glial cell line-derived neurotrophic factor (GDNF) are good materials for the delivery of cellular drugs and the treatment of PD [235]. RNAi-loaded plasmid nanoparticles achieved effective repair in a PD model (Fig. 11A) [236]. Exosomes derived from human endometrial stem cells inhibit S protein aggregation and prevent neuronal cell death [217].

Traumatic Brain Injury (TBI) is a highly morbid brain tissue damage disease, and the number of deaths caused by it is increasing each year. Oxidative stress and local inflammation at the site of TBI damage are key factors determining the outcome of the treatment, and SHED-Exo is able to effectively ameliorate the inflammation in the neural tissues and promote the repair of TBI tissues [237]. However, instability, intermittency, and induction of oxidative stress during Exo release can exacerbate damage to the original site. Li et al. [191] modulated TBI nerve repair by preparing a hybrid hydrogel based on poly(citrate-gallic acid) (FPGEGa) by hydrogel modification and conjugation with microglia. FPGEGa exhibited self-healing, thermo-sensitizing, and antioxidant effects, and the sustained release of SHED-Exos reduces ROS levels in the central nervous system. The hydrogel can also induce microglia to exhibit stronger anti-inflammatory effects by promoting anti-inflammatory M2 polarization and inhibiting pro-inflammatory M1 polarization, providing a new therapeutic strategy for TBI and other tissue engineering fields (Fig. 11B).

Gene therapy hydrogels may also treat other brain diseases, such as Alzheimer’s disease and Huntington’s disease. These diseases are often associated with amyloid deposits and genetic mutations. Gene therapy drugs for these diseases can be delivered directly to the brain to promote nerve cell repair and regeneration with the use of gene therapy hydrogels.

### Enteric diseases

Enteritis diseases are inflammatory diseases of the intestines, including infectious and non-infectious enteritis, and the symptoms of this type of disease include diarrhea, abdominal pain, nausea, and vomiting [238]. Oral administration of siRNA-containing nanolipids is a highly effective siRNA delivery method to treat ulcerative colitis (UC) [239]. The diagnosis of enteritis requires tests. Routine stool examination is a basic procedure that can detect white blood cell and red blood cell indicators in the stool. Enteroscopy is a method used to visually inspect the intestinal mucosa, directly observe changes, and obtain tissue samples for pathological examination. Furthermore, blood tests, radiological imaging, and immunological tests are often necessary. Infectious enteritis is mainly treated with antibiotics and other medications, hydration, and electrolytes, whereas non-infectious enteritis needs to be treated with immunomodulators, hormones, and other medications, and sometimes with surgery [238].

Ulcerative colitis is the main type of inflammatory bowel disease that causes diarrhea, blood in the stool, colon atrophy, and colorectal cancer. Daily oral medications only provide short-term relief from the inflammatory response, and biologics in the antibody drug class were effective in suppressing colitis. However, this requires large doses of antibodies, has low bioavailability, and triggers systemic toxicity [240]. In contrast, siRNAs are significantly more valuable because they are more efficient, non-toxic, and safer. It is difficult for siRNAs to exert therapeutic effects on UC by oral administration owing to their degradation in the gastrointestinal environment [241]. Gao et al. [192] designed a SA@MOF-siRNATNFα drug delivery system to avoid oral degradation. The stability of the system was demonstrated in the acidic environment of the small intestine and its successful uptake by inflammatory macrophages, resulting in increased siRNATNFα release. Furthermore, siRNA had a concentration and infiltrating effect, which led the system to reduce the severity of enteritis more efficiently and provided a prospective therapeutic option for treating the colon.

### Rheumatoid arthritis

Rheumatoid arthritis (RA) is a systemic inflammatory disease whose etiology is not yet clear, with mostly symmetric, chronic, multi-synovial arthritis, extra-articular lesions as clinical manifestations. RA occurs in the small joint tissues of the feet, wrists, and hands, and is often recurrent, with red, swollen joints and hot pain at early stages, and stiff and deformed joints at late stages, accompanied by atrophy of the bones and skeletal muscles. Damaged tissues are easily disfigured and disabled if not treated in a timely manner [242]. siRNA is commonly used to treat RA and specifically silences genes involved in the pathogenesis of RA. Specific silencing of genes involved in the pathogenesis of RA can be achieved by spraying arthritic mice with siRNA-containing hydrogels [29]. Kanazawa et al. [29] used sericin hydrogels to load anti-RelA siRNA hydrogels to achieve sustained-release siRNA in vitro, and the knee joints of the rats were continuously degraded for a long time in vivo. CIA mice treated with a silk glycoprotein hydrogel containing anti-RelA siRNA showed significant improvements in morbidity, knee joint thickness, and clinical severity compared to controls.

Exosomes are small membrane vesicles produced by cells and released outside the cell; they contain proteins, nucleic acids, and lipids and are widely recognized as important carriers of information transfer between cells [115, 243]. Exotherapy is a recognized and highly promising strategy for RA treatment; however, it still presents serious challenges in the long-term modulation of the specific pathogenesis of RA and the protection of reduced joint damage. Therefore, Rui et al. [193] prepared a photo-cross-linked SF hydrogel and encapsulated olfactory ecto-MSC-derived Exos to form the Exos@SFMA hydrogel for continuous treatment of the immune microenvironment of RA. Hydrogels have flexible mechanical properties, excellent biocompatibility, and adsorption, and are suitable for the protection of joint tissue surfaces. Furthermore, the hydrogel promotes PD-L1 expression, which inhibits the PI3K/AKT pathway to achieve inhibition of polarization of T-follicular helper cells (Tfh). The hydrogels inhibited the differentiation of germinal center B cells into plasma cells by decreasing the Tfh cell response, which prevented joint damage and alleviated synovial inflammation. In summary, gene therapy hydrogels are an efficient and effective therapeutic strategy for RA with great potential for clinical applications.

### Hair regeneration

**Fig. 12:**
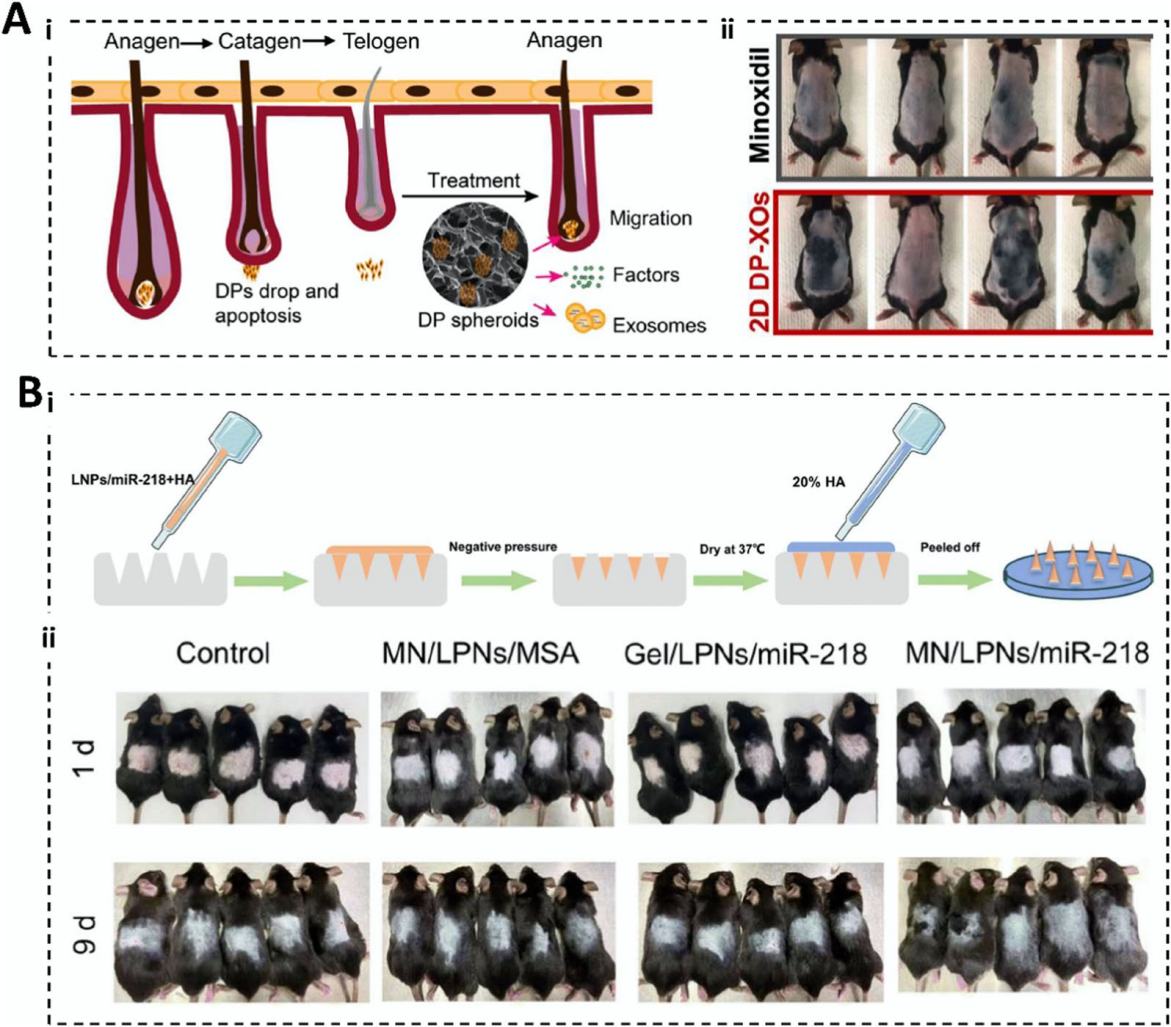
Applications of therapeutic gene hydrogels in hair regeneration. **A** miR-218-5p-containing exosomes modulate the β-catenin signaling pathway to promote hair regrowth. (i) Schematic representation of dermal papilla globules promoting hair by secreting exosomes; (ii) Exosome hydrogel promotes back hair regeneration in mice. (Reproduced with permission from Ref. [246], Copyright 2020, American Association for the Advancement of Science). **B** LPN microneedle patch delivers miR-218 to promote hair regrowth. (i) Schematic representation of the preparation of MN/LPNs/miR-218; (ii) Schematic representation of hair regeneration in mice administered at different times. (Reproduced with permission from Ref. [156], Copyright 2022, Royal Society of Chemistry)

Hair regrowth specifically stimulates hair growth, increasing hair density and length. Hair regeneration is achieved through the activation and differentiation of stem cells to form newborn hair cells, which are then transformed into hair fibers [244]. Some growth factors (such as hepatocyte growth factor, insulin-like growth factor, vascular endothelial growth factor, platelet-derived growth factor, and transforming growth factor beta) promote hair regrowth. They promote hair regeneration by stimulating hair follicle cell proliferation, improving the follicular periphery and vascular microenvironment, inhibiting apoptosis, and repairing endothelial damage. However, excessive growth factors can induce adverse effects [245]. Many approaches were used for hair regeneration, with cell therapy and tissue engineering being the most promising. Exosomes with internal miR-218-5p promote hair regeneration by modulating β-catenin signaling (Fig. 12A) [246]. Cell therapy involves autologous or allogeneic cells, such as hair follicle stem cells and fibroblasts for hair regeneration. Tissue engineering involves the use of biomaterials and stem cells to build hair structures that are then transplanted, and these methods have yielded remarkable results [247].

Tao et al. [194] stimulated human dermal microvascular endothelial cells (HMEC-1) and human dermal fibroblasts stimulated proliferation in a dose-dependent manner in miR-126-3p overexpressing SMSCs (SMSC-126-Exos). miR-218 down-regulates SFRP2 to activate the Wnt/β-catenin channel, which is a key channel in the transformation of the follicular cycle for hair regeneration. Therefore, it can achieve hair regeneration by delivering the miR-218 gene. However, miRNA targeting the target site is limited owing to skin barrier restrictions (such as stratum corneum obstruction) or insufficient resistance of miR-218 to enzymatic degradation. Zhao et al. [156] used HA microneedles to enhance the permeability of the stratum corneum and then used lipid polymer hybrid nanoparticles (LPNs) as the delivery vehicle of miR-218 to avoid enzymatic degradation, which efficiently promoted the diffusion of LPNs/miR-218 in the dermal region and maximized the utilization of miR-218 (Fig. 12B). In conclusion, this system enables the targeted delivery of miRNAs for hair regeneration.

Gene therapy hydrogels have numerous applications for tissue engineering. First, they can function as a gene carrier to deliver therapeutic genes accurately to target organs, enhance the effects of gene therapy, and minimize side effects. Second, they promote cell proliferation for tissue repair. Finally, they can control the rate and mode of gene release to optimize the efficacy and repair of damaged tissues. Despite its superior biocompatibility, the hydrogel can cause immune and inflammatory reactions. Some hydrogels are difficult to degrade naturally, and this may cause tissue damage. Bone remodeling can be achieved through autografts and xenografts, but it is difficult owing to its adverse immune response, potential risk of infection, and restricted sources. Some hydrogels have shortcomings such as low mechanical strength, an inhomogeneous crosslinking structure, slow response, and low crosslinking density. Furthermore, the ease of degradation of therapeutic genes, stability, and safety issues also affect the final efficacy. Therefore, measures such as the introduction of biologically active components, the improvement of the preparation process, and the development of novel crosslinking agents are needed to overcome these problems.

## Summary and future directions

Delivery strategies targeting tissue have received significant attention in recent decades owing to their ability to effectively avoid complications associated with therapeutic gene delivery. Hydrogels that serve as carriers of therapeutic genes exhibit excellent biocompatibility, biodegradability, and solubility. These properties shield therapeutic genes from enzymatic degradation and microenvironmental effects, facilitating sustained and slow gene release and enhancing therapeutic outcomes. Hydrogels can also enable responsive drug release through functional modifications. Gene therapy hydrogels find widespread application in bone, heart, trauma, cartilage, spinal cord, skin, eyes, and tumors, demonstrating robust scientific and clinical efficacy across diverse tissues and organs.

Although gene therapy hydrogels offer considerable advantages, they face several challenges. Natural polysaccharide hydrogels exhibit inadequate control, mechanical properties, and permeability. Furthermore, the degradation products of natural polysaccharide hydrogels can incite immune reactions. Protein hydrogels suffer from insufficient degradation rates, bioactivity, and stability, and are susceptible to external pH and temperature influences. Despite the chemical stability and controllability of synthetic hydrogels, issues like poor biocompatibility, slow degradation, and biotoxicity persist and cause resolution. Moreover, optimizing the crosslinking of hydrogels remains an ongoing challenge. Therapeutic genes are prone to degradation, instability, mutations, and safety concerns, demanding continuous refinement and modification. While ZFNs, TALENs, and CRISPR-Cas9 modifications have found wide application in gene therapy, their limitations require further improvement. The loading of therapeutic genes into hydrogels presents both advantages and disadvantages, requiring enhancement.

Certain gene therapy hydrogels exhibit inadequate mechanical properties and stability, potentially triggering immune reactions and toxic effects. Consequently, safety and efficacy are primary considerations. Furthermore, poor transfection efficiency can hinder the efficient delivery of gene drugs and all these factors affect tissue engineering applications. Gene therapy, as a therapeutic means of intervening directly against genetic defects or abnormalities, is of great potential therapeutic efficacy and clinical value. Before gene therapy is carried out, the treatment strategy needs to be comprehensively evaluated and optimized to ensure the safety and effectiveness of the treatment process. Secondly, the safety of gene therapy also needs to be ensured through strict clinical trials and regulation. However, we must also be aware that there are still challenges and uncertainties regarding the safety of gene therapy. For example, the assessment of long-term therapeutic effects and potential side effects needs to be supported by longer and larger-scale clinical studies; issues such as immune rejection and genetic instability that may be triggered by gene therapy also need to be further researched and resolved.

In summary, gene therapy hydrogels have shown significant advantages in tissue engineering and play an active role in a wide range of organs and tissues. However, its application still faces numerous areas for improvement and requires continuous exploration and effort. With a deeper understanding of gene function and regulatory mechanisms, gene therapy strategies will become more precise and personalized. This means that future gene therapy hydrogels will need to be able to precisely deliver therapeutic genes to target cells or tissues while achieving fine regulation of gene expression levels. Future research should be devoted to optimizing material types, improving cross-linking methods, and broadening gene modification methods in order to continuously improve the key properties of gene therapy hydrogels, such as mechanical properties, biocompatibility, and transfection efficiency. Moreover, with the deepening of crossovers between disciplines, we believe that more advanced and effective gene therapy hydrogels and tissue engineering products can be developed. The close integration of gene therapy and hydrogels will bring brand new possibilities to the field of tissue engineering, inject new vitality into materials science, provide more effective means of clinical treatment, and bring better therapeutic effects and quality of life to patients.

## Data Availability

The data used to support this review are included within the article.
